# Plexin B1 controls Treg numbers, limits allergic airway inflammation, and regulates mucins

**DOI:** 10.3389/fimmu.2023.1297354

**Published:** 2024-01-08

**Authors:** Svetlana P. Chapoval, Hongjuan Gao, Rachel Fanaroff, Achsah D. Keegan

**Affiliations:** ^1^ Department of Microbiology and Immunology, University of Maryland School of Medicine, Baltimore, MD, United States; ^2^ Center for Vascular and Inflammatory Diseases, University of Maryland School of Medicine, Baltimore, MD, United States; ^3^ Program in Oncology at the Greenebaum Cancer Center, University of Maryland School of Medicine, Baltimore, MD, United States; ^4^ Department of Physiology, University of Maryland School of Medicine, Baltimore, MD, United States; ^5^ Department of Pathology, University of Maryland School of Medicine, Baltimore, MD, United States; ^6^ Veteran Affairs (VA) Maryland Health Care System, Baltimore Veteran Affairs (VA) Medical Center, Baltimore, MD, United States

**Keywords:** semaphorin 4, plexin B1, Tregs, allergy, inflammation

## Abstract

We investigated the effect of global Plexin B1 deficiency on allergic airway responses to house dust mite (HDM) or ovalbumin (OVA). In the HDM model, there were higher Th2 cytokine levels in the BALF of Plexin B1 knock-out (KO) mice compared to wild type (WT), and tissue inflammation and mucus production were modestly enhanced. In the OVA model, Plexin B1 deficiency led to increases in lung inflammation, mucus production, and lung Th2 cytokines accompanied by dysregulated mucin gene expression without affecting anti-OVA IgE/IgG1 levels. Spleen cells from Plexin B1 KO mice proliferated more robustly than WT cells *in vitro* to a variety of stimuli. Plexin B1 KO CD4+ T cells from spleens expressed higher levels of Ki-67 and CD69 compared to WT cells. Spleen cells from naïve Plexin B1 KO mice secreted increased amounts of IL-4 and IL-6 when pulsed *in vitro* with OVA whereas *in vivo* OVA-primed spleen cells produced IL-4/IL-5 when subjected to *in vitro* OVA restimulation. The upregulated allergic inflammatory response in Plexin B1 KO mice was associated with a lower number of Tregs in the lung tissues. Moreover, these mice displayed lower numbers of Treg cells in the lymphoid tissues at the baseline. These results demonstrate a previously unrecognized link between Plexin B1, Treg cells, and mucus in allergic lung inflammation.

## Introduction

Asthma is a chronic inflammatory lung disease that is characterized by airway inflammation, mucus hypersecretion, smooth muscle hypertrophy, and oedema of the airway wall leading to airway narrowing (1). Asthmatic lung tissue is infiltrated with inflammatory cells with notable upregulation of Th2 cytokines in a major asthma Th2 endotype (1, 2). Foxp3+ regulatory T cells are potent suppressors of Th2 cell activation and Th2-driven lung inflammation (3). Therefore, the *in vivo* stimulation of Treg cells can provide a beneficial immunosuppressive effect in overexaggerated asthmatic lung response (3–6). Our current study focuses on the stimulatory effect of neuroimmune semaphorin 4A (Sema4A) on Treg cells by utilizing its receptor Plexin B1 (7, 8).

Plexin B1 (alternative names: PLXNB1, Semaphorin receptor SEP, KIAA0407, PLXN5, SEP) serves as a receptor for class 4 semaphorin family proteins, Sema4A and Sema4D (9–13). The role of Plexin B1 in autoimmune diseases, cancer, and cardiovascular diseases has been extensively studied and mostly associated with its engagement of Sema4D (14–19). The role of Plexin B1-Sema4A interaction in disease is less defined (10, 12, 20). One report shows that the Sema4A-Plexin B1 pathway plays a critical role in the directional guidance of axonal elongation during brain development and growth cone collapse in mouse hippocampal neurons *in vitro* (20). Wang and associates (12) assessed the significance of Sema4A-Plexin B1 pathway in the inflammatory response in rheumatoid arthritis (RA) by using human synovial fibroblasts of RA (RASFs) in *in vitro* cultures. When RASFs were stimulated with rSema4A, they secreted a substantial amount of IL-6 which was almost completely abrogated by siRNA to Plexin B1. The authors concluded that an increased expression of Sema4A found in synovial fluid of patients with RA is required to promote inflammation.

Sema4A is known to act as an axon guidance molecule in the nervous system; in the immune system it can act as a costimulatory molecule for T cell activation and a negative regulator of tissue inflammation (10, 21–23). We previously showed that Sema4A deficient mice displayed enhanced allergic airway inflammation accompanied by fewer Treg cells, however the. identity of the Sema4A counter receptor(s) responsible for this effect was unknown (10, 23). We subsequently reported that Sema4A exerts a stabilizing effect on human Treg cells in PBMC and CD4+ T cell cultures by engaging Plexin B1 (8). While Sema4A deficient mice displayed enhanced allergic airway inflammation accompanied by fewer Treg cells, Sema4D deficient mice displayed reduced inflammation and increased Treg cell numbers even though both Sema4 subfamily members can engage Plexin B1 (10, 23, 24). Moreover, Sema4A and Sema4D display opposite effects on human Treg cells in *in vitro* cultures of peripheral blood mononuclear cells (PBMC); Sema4D inhibited CD4+CD25+Foxp3+ cell numbers and CD25/Foxp3 expression (7).

Therefore, to determine the role of Plexin B1 in allergic inflammation, we generated and characterized responses to allergens in mice lacking Plexin B1, Plexin B1knock-out (KO). We observed that mice systemically lacking Plexin B1 have a lower pool of Treg cells in lymphoid organs and lungs as compared to wild type (WT) mice. Additionally, Plexin B1 was found to control selected mucin gene expression. The *in vivo* Plexin B1 deficiency resulted in heightened allergic airway inflammatory response and mucin dysregulation in response to allergen exposures. These results will be informative for the future design of Sema4A-Plexin B1-based therapeutics for allergic asthma, given that Sema4A functions as a downregulatory molecule for allergen-induced disease in mice (23).

## Materials and methods

### Mice

C57BL/6J mice (WT mice) were obtained from Jackson Laboratory. Plexin B1 KO mice (**Supplementary Figure S1**) were generated at Cyagen (KOCMP-235611-*Plxnb1*-B6J-VA, Santa Clara, CA; C57BL/6J-*Plxnb1* ^em1cyagen^). In brief, the *Plxnb1* gene encoding Plexin B1 was altered via Crispr/Cas9 gene targeting in embryonic stem cells. Homologous recombination led to deletion of exons 4-16 of the *Plxnb1* gene (**Supplementary Figure S1**). The deletion was identified via PCR with a forward primer (F1) annealing 5’ to exon 4 and reverse primer (R1), that anneals 3’ to exon 16. Deletion in F1 animals was confirmed by Cyagen by sequencing which demonstrated a deletion of 5524 bp of mouse *Plxnb1* and a 135 bp insertion. Heterozygous mice were inter-bred to generate homozygous targeted mice. The homozygous Plexin B1 KO mice were bred at the University of Maryland School of Medicine by brother sister mating and genotyped to confirm homozygosity of the targeted *Plxnb1* allele using the following primer sets:

Primer’s set 1: (Annealing Temperature 60.0 °C)

F1: 5’-AATATAGGAGTGAGCATTGGAGCA-3’

R1: 5’-TCCAGAGTGTTAAGTCTACAGTGG-3’

Product size: KO 610 bp; WT 5999 bp

Primer’s set 2: (Annealing Temperature 60.0 °C)

F1: 5’-AATATAGGAGTGAGCATTGGAGCA-3’

R2: 5’-CATACCCTATGCAACTGTCCTTGA-3’

Product size: 588 bp

Homozygotes Plexin B1 KO display one band of 610 bp (using F1, R1 primers), while wild type alleles display one band of 588 bp (using F1, R2 primers). WT and Plexin B1 KO (C57BL/6J-*Plxnb1* ^em1cyagen^) mice were maintained under specific pathogen-free conditions with free access to food and water. All experiments with the mice were performed in compliance with the principles and procedures outlined in the NIH Guide for Care and Use of Animals and were approved by the University of Maryland School of Medicine Animal Care and Use Committee.

### Anesthetic

Avertin in dose of 0.3 mg/kg or 2 mg/kg by i.p. injection was used as previously described (25) to anesthetize or euthanize the mice, correspondingly.

### Kidney size measurements

Analysis of kidney size was performed as described previously (26). Left and right kidneys were dissected from 13-week-old male mice. The kidney length and width were measured in mm and overall kidney area was calculated as length x width (mm2).

### Experimental protocols

a) House dust mite (HDM)-induced allergic response. WT and Plexin B1 KO mice were treated with HDM allergenic extract (lot no. 360924, Greer Laboratories, endotoxin concentration: 13.14 EU/50 μg HDM dose) as previously described (27). Briefly, 50 μg of extract in 5̃ μl PBS was applied to anesthetized mice intranasally four times six days apart. The resulting inflammatory response was assessed 48h after the last HDM application.

b) OVA-induced allergic response. WT and Plexin B1 KO mice were immunized with OVA (Endofit OVA, low-endotoxin concentration; InVivoGen Labs) adsorbed to Alum as described previously (25, 28, 29). Briefly, mice were immunized with either 100 μg of OVA/Alum or Alum alone by IP injection on day 1 and again on day 6. Mice were challenged with aerosolized PBS or 1% OVA in PBS for 40 minutes each day on days 12 and 14. The assessment of allergic lung response was performed 48h later.

### Bronchoalveolar lavage collection and cell composition

BALs were obtained from euthanized mice and processed as previously described (28, 29). Cytospin preparations were made with 200 μL of BAL fluid (Cytospin 2; Shandon Inc., Pittsburgh, PA) and stained with Diff-Quick (Dade Behring, Deerfield, IL). The differential cell counts were determined from 4 high-power fields.

### Histochemistry and score-based quantification of lung histology

Lungs were obtained from euthanized WT and Plexin B1 KO mice after completion of the allergen treatment protocols and prepared as previously described (27, 30). N = 3-7 mice per experimental group in 3-4 independent experiments were used. Lung sections were processed at the University of Maryland School of Medicine’s Pathology Histology core within the Center for Innovative Biomedical Resources (CIBR) and prepared for histochemistry (H&E and PAS stains) of deparaffinized lung tissues. Lung sections were evaluated by an investigator blinded to the experimental conditions. H&E and PAS stains were evaluated as previously described (27, 30): for H&E, 0, no signs of inflammation; 1, light and dispersed infiltrate in only a few areas of the section; 2, modest infiltrate surrounding major vessels and airways, with little in distal airway unit; 3, moderate infiltrate surrounding <50% of distal airways and vessels; and 4, heavy and focused eosinophilic infiltrate surrounding numerous distal vessels and airways, signs of increased alveolar macrophage numbers with macrophage fusions and extracellular matrix deposition. For PAS: 0, no signs of mucus in airways or elevated numbers of PAS+ cells; 1, no mucus in airways, slight increase in numbers of PAS+ cells in a few major airways; 2, some mucus detectable in major airways; 3, mucus detected in major airways with <50% of distal airway epithelial cells PAS+ in distal airway units; and 4, mucus plugging of airways observed in several airways and majority of distal airway epithelial cells are PAS+ in multiple distal airways per section.

### Goblet cell hyperplasia

Goblet cell hyperplasia was evaluated on PAS-stained lung sections. For observation consistency, similarly sized PAS+ airways were identified on each slide and digitally imaged at 20X magnification. PAS+ cells were quantified from two-three images per sample using ECHO imaging system. PAS+ cells were counted manually and normalized to the length of the basement membrane (31).

### Cytokines, chemokines, Mucin 5AC, and soluble Sema4A in BALF

The levels of BALF cytokines and chemokines were determined using Searchlight Proteome Array (Aushon) at the Cytokine Core Lab, Center for Innovative Biomedical Resources, University of Maryland School of Medicine. The array data were analyzed using the ArrayVision software. In studies where the absolute value of BAL cytokines varied in independent experiments, cytokine values were normalized to allergen-induced fold increase relative to the value of control PBS-treated WT mice (set as 1). The Muc5AC concentrations in BALF were determined with the use of ELISA kit NBP2-76704 (Novus Biologicals) according to the manufacturer’s instruction. Mouse Sema4A ELISA kit (MBS3807413, MyBioSource) was used to measure the levels of soluble Sema4A in BALF at 48h after last OVA challenge.

### WST-1 cell proliferation assay

The Premix WST-1 Cell Proliferation Assay System (#MK400, Takara) was used to assess the number of metabolically active cells, a surrogate for cell proliferative responses *in vitro*. Single cell suspensions were prepared from spleens of naïve, or PBS- and OVA-injected mice on day 5 post-treatment. Spleen mononuclear cells (MNC) were plated to a density of 1 × 10^6^ cells/200μl in 96-well tissue culture plates (#353072, Falcon) and stimulated with either Concanavalin (Con) A (10μg/ml; Invitrogen), LPS (100μg/ml; #L5293, Sigma-Aldrich), OVA (from 0.001 to 100 mg/ml; InvivoGen), or OVA_323–339_ peptide (200μg/ml; #RP10610, GenScript) as previously described (23). After 72h of incubation, 20μl of tetrazolium salt WST-1 solution was added to each well followed by 4h of further incubation. The stable tetrazolium salt is cleaved to soluble formazan by a process dependent on the glycolytic production of NAD(P)H in viable cells which directly correlates to the number of metabolically active cells in the culture. The plates were read in the ELISA plate reader at 450 nm with a reference wavelength of 650nm.

### *In vivo* proliferation assay and measurement of T cell activation

WT and Plexin B1 KO mice were injected with OVA/Alum/200 μl and 1 mg/100 μl BrdU i.p. on day 0 (9). Mice received BrdU (EMD Millipore, #19-160) injection the following day. Twenty-four hours later mice were sacrificed, spleens were harvested and processed to single cell suspensions. Spleen MNC were stained with Abs to cell surface markers (CD4 and CD69) and BrdU expression was analyzed by flow cytometry employing FITC BrdU Flow kit (BD Pharmingen, #51-2354AK) for staining according to the manufacturer’s instruction.

### Flow cytometry

Flow cytometry of lung, lymph node, and spleen single cell suspensions was performed as previously described (30, 32, 33) using the BD Biosciences Abs for the following cell markers: I-Ab (#562823, APC), CD3 (#553061, FITC), CD4 (#563933, PE-Cy7), CD8 (#553036, PerCP), CD25 (APC, #557192), and CD45R/B220 (#552771, PerCP-Cy5.5). Intracellular staining for Foxp3 was done using anti-Foxp3 Ab or rat IgG2a(kappa) isotype control Ab (both AlexaFluor700 and from eBioscience, #56-5773-80 and #56-4321-80, correspondingly) with mouse Foxp3 Buffer Set (#560409, BD Biosciences). For detection of activation marker expression on CD4+ T cells, spleen MNC were cultured *in vitro* in medium (cRPMI) alone, OVA (20 μg/1 x 10^6^ cells/well), or ConA from Invitrogen (500X, #00-4978-93; 2 μl/1 x 10^6^ cells/well) or from Sigma (C5275, 0.028 μg/well) for 36h. The cells were harvested, stained, and analyzed for CD69 expression using anti-CD69 PE-labeled Ab from Invitrogen (#12-0691-81) or armenian hamster IgG PE (# 12-4888-81) as an isotype control Ab, or for Ki-67 intracellular expression using mAb FITC (clone SOL815) from Termofisher Scientific with rat IgG2a FITC in control tubes. For dead cell discrimination in flow the LIVE/DEAD™ Fixable Aqua Dead Cell Stain Kit was used (Invitrogen). For the assessment of Plexin B1 expression on spleen and lymph node T cells, single cell suspensions were stained with anti-Plexin B1 primary Ab (clone A-8, Santa Cruz) followed by mouse-IgGκ BP-CFL 488 (sc-516176, Santa Cruz) or anti-mouse IgG-APC (#F0101B, R&D Systems). The cells in the control tubes were stained with purified mouse IgG2B (MAB0041, R&D Systems) followed by a secondary fluorochrome- labeled Ab. Cells were pre-treated with purified rat anti-mouse CD16/CD32 (Mouse BD Fc Block) Ab before staining for specific markers. Cells gated by forward- and side-scatter parameters were analyzed using the FlowJo 10 software.

### RT-qPCR

Total lung RNA was extracted using TRIzol Plus RNA Purification Kit (#12183555, Invitrogen) (34). Briefly, the kidney or lung tissues samples were placed in 1 ml of TRIzol reagent and homogenized using immersion homogenizer (Omni International, Southern Labware, Cumming, GA). Chloroform and 4-bromoanisole additions were used for lysis, and aqueous solution with RNA was treated with ethanol for precipitation. The PureLink RNA Mini Kit (Invitrogen) was used for RNA membrane-binding and elution. iScript gDNA Clear cDNA Synthesis Kit (Bio-Rad) was used for the first strand DNA syntheses at Bio-Rad T100 Thermal Cycler and PowerUp SYBR Green Master Mix (Applied Biosystems) was used for qPCR workflow on QuantStudio 3 (Applied Biosystems) with sets of primers for mucins and *Plxnb1*, exons 11 and 38 (17, 35–38) shown in **Supplementary Table 1** (Integrated DNA Technologies). The expression of the target gene was calculated using the ^2−ΔΔ^Ct method, and the expression level was relative to Hypoxanthine Phosphoribosyltransferase 1 (HPRT1) as the reference gene. RNA and DNA concentrations were measured using NanoDrop One spectrophotometer (ThermoScientific).

### Total IgE and anti-OVA IgE/IgG1 in sera

Total and OVA-specific serum IgE/IgG1 Ab levels at different time-points during the allergen immunization protocol were measured using Mouse IgE ELISA kit (Invitrogen), Mouse anti-OVA IgE assay kit (Chondrex), and anti-Ovalbumin IgG1 ELISA kit (Cayman Chemical), correspondingly. All ELISA plates were read on either iMark (Bio-Rad) or SpectroStar Nano (BMG Labtech) microplate reader using the manufacturer specified wavelengths for each assay.

### Statistics

Data were summarized as mean ± SEM. To calculate significance levels between experimental groups, two-way ANOVA with Tukey’s multiple comparison test, ordinary one-way ANOVA, and non-parametric Mann-Whitney’s test (GraphPrizm8) were performed as indicated in the corresponding figure legends.

## Results

### Generation and characterization of Plexin B1 KO mice

We reported previously that Plexin B1 plays a critical role in human Treg cell stability and function *in vitro* (7, 8). To investigate the role of Plexin B1 signaling in Treg cell stability and function *in vivo*, Plexin B1 KO mice were generated at Cyagen (**Supplementary Figure S1A**). Plexin B1 KO mice were found to be viable and fertile. They displayed an increase in kidney size measured at post-gestational week 13 compared to kidneys obtained from age-sex matched WT mice consisted with a previous study (26) on the role of Plexin B1 in kidney morphogenesis (**Supplementary Figure S1B**). They demonstrated a lack of Plexin B1 transcripts in kidney, a tissue known to express high levels of Plexin B1 (**Supplementary Figure S1C**). We further evaluated Plexin B1 transcripts in the lungs of mice treated with allergenic stimulations, either OVA/Alum or HDM as indicated (**Supplementary Figures S1D, E**). OVA treatment increased lung Plexin B1 expression over the PBS control while HDM treatment reduced Plexin B1 in WT mice. Plexin B1 transcripts were not detected in lungs isolated from Plexin B1 KO mice using primer pairs located in exon 11 or exon 38.

### Plexin B1 modulates multiple parameters of allergic inflammation

**Figure 1:**
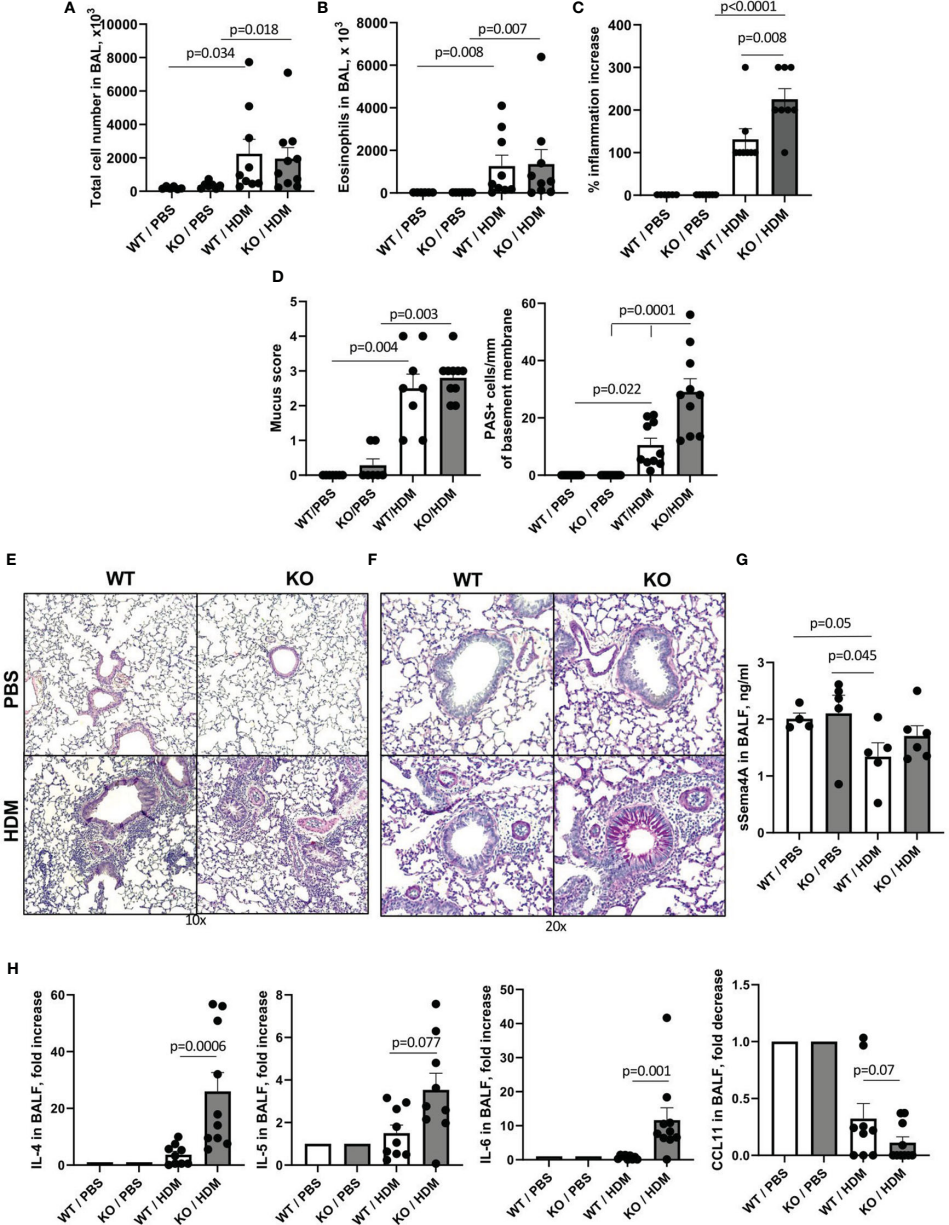
Effect of Plexin B1 deficiency on HDM-induced allergic lung inflammation and mucous cell hyperplasia in mice. WT and Plexin B1 KO mice were treated with HDM as described in Materials and Methods. BAL was performed and evaluated for total number of cells **(A)** and eosinophil numbers **(B)**. Lung sections were stained with H&E or PAS; the slides were scored for inflammation **(C)** and overall PAS staining and numbers of mucous cell hyperplasia **(D)** as described in Materials and Methods. Representative photomicrographs of lung tissue stained with H&E **(E)** and PAS **(F)** are shown. Levels of soluble Sema4A **(G)** and cytokines **(H)** in BALF were evaluated as described in Materials and Methods. Data from two independent experiments are shown (n=4-5 mice/group) and expressed as fold-increase over a PBS-treated WT control. Data statistics were calculated using two-way ANOVA with Tukey’s multiple comparison test, two-tailed Mann-Whitney’s non-parametric test, and Wilcoxon’s non-parametric paired test.

We examined the response of WT and Plexin B1 KO mice to house dust mite (HDM), a common human allergen highly associated with allergic asthma and disease exacerbation. To detail Plexin B1 function in the HDM-induced allergic response, WT and Plexin B1 KO mice were subjected to four intranasal applications of HDM allergenic extract six days apart. Control mice were treated with endotoxin-free PBS. A classical allergic airway response was observed in WT mice 48h after the last HDM application (**Figures 1A-C**). This response consisted of prominent BAL (**Figure 1B**) eosinophilic infiltration where more than 50% of BAL cells were eosinophils. HDM also stimulated multiple peribronchial and perivascular inflammatory infiltrates (**Figure 1E**). The allergic inflammatory response was more pronounced in the lung tissues of Plexin B1 KO mice as the inflammation score was significantly higher than that in WT mice (**Figure 1C**). Although the overall mucus score did not differ significantly between WT and KO mice, the number of hyperplastic mucous cells per mm of basal lamina was significantly upregulated by Plexin B1 deficiency (**Figure 1D**). Levels of soluble Sema4A in BALF did not differ between untreated WT and Plexin B1 KO mice (**Figure 1F**). However, HDM treatment led to a downregulation of BALF Sema4A as compared to PBS-treated controls in WT mice but not in Plexin B1 KO mice. The relative levels of Th2 cytokines IL-4 and IL-5 in BALF (**Figure 1H**) were increased in Plexin B1 KO mice, however, the increase in IL-5 did not achieve statistical significance. IL-13 levels in BALF were below the limit of assay detection. IL-6 levels were significantly upregulated in HDM-treated Plexin B1KO mice (**Figure 1H**) while CCL11 levels were reduced (**Figure 1H**). This paradoxical decrease in CCL11 may be due to its consumption by the increased number of eosinophils. No significant changes in total TGFβ and IL-10 levels in BALF between PBS- and HDM-treated WT and KO mice were detected (data not shown). BALF TNFα levels were below the limit of detection (19 pg/ml). Thus, Plexin B1-deficiency resulted in enhanced features of HDM-induced experimental allergic airway inflammation.

**Figure 2:**
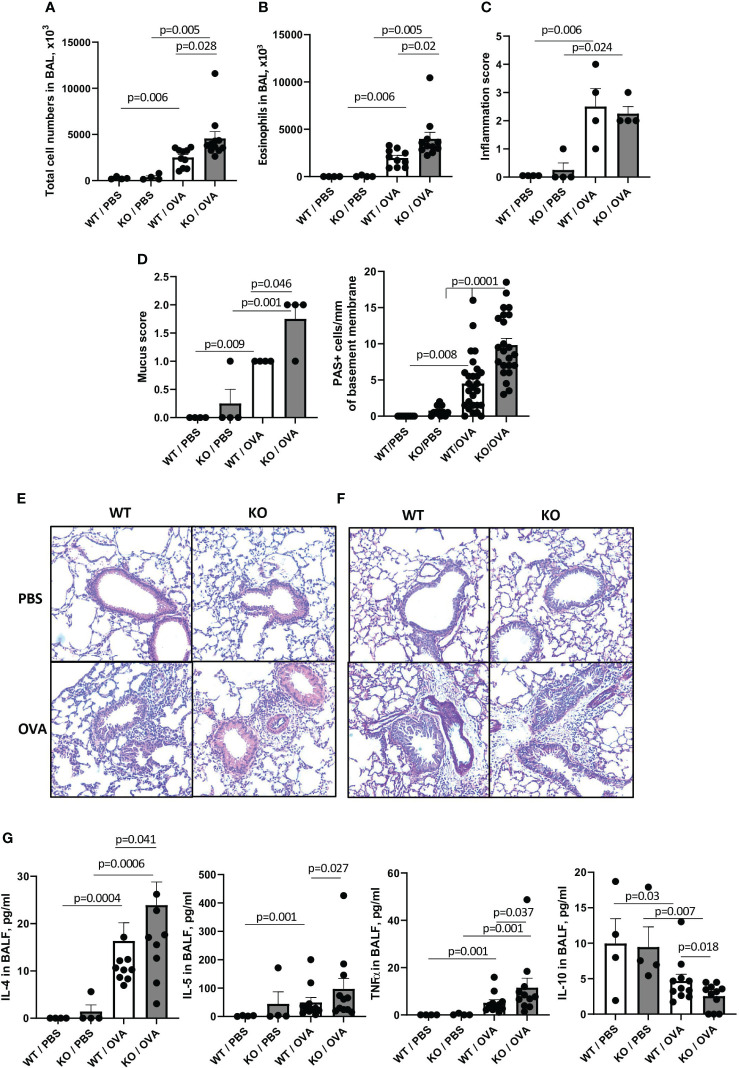
Effect of Plexin B1 deficiency on inflammation, mucus production, and cytokine levels in BALF after OVA treatment. WT and Plexin B1 KO mice were immunized with alum or OVA/alum and challenged as described in Materials and Methods. BAL was performed and evaluated for total number of cells in BAL **(A)** and eosinophil numbers **(B)**. Lung sections were stained with H&E or PAS and scored for levels of inflammation **(C)** or overall PAS staining and numbers of mucous cell hyperplasia **(D)** as described in Materials and Methods. Representative photomicrographs for H&E **(E)** and PAS **(F)** are shown. **(G)** Levels of cytokines in BALF were evaluated as described in Materials and Methods. Data from two independent experiments are shown (n=4-11 mice/group). Data statistics were calculated using two-way ANOVA with Tukey’s multiple comparison test, two-tailed Mann-Whitney’s non-parametric test, and Wilcoxon’s non-parametric paired test.

Since most of the previously published studies on the effects of Sema4A and Sema4D deficiencies an allergic inflammation used the classic Ova/alum model (23, 24), we also compared the responses of WT and Plexin B1 KO mice to the classic OVA/Alum prime, boost, and OVA challenge as described in the Materials and Methods section. Allergen exposure of WT mice led to significant increases in BAL total cell numbers from 261.0 x10^3^ cells in PBS-treated mice to 2511.0 x 10^3^ cells in OVA-treated mice (**Figure 2A**). Eosinophilia was also elevated from 0 eosinophils noted in PBS-treated mice to 1963.0 x 10^3^ after OVA exposure (**Figure 2B**). However, similar to what we observed in Sema4A-deficient mice (23), these increases were even more prominent in Plexin B1 KO mice, reaching to 4573.0 x 10^3^ total cells and 3993.0 x10^3^ eosinophils after OVA exposure. The absolute number of lymphocytes in BAL was equal between OVA-treated WT and KO mice amounting to 243.3 x 10^3^ and 273.1 x 10^3^), correspondingly. Although the overall lung inflammation scores were not significantly different between OVA-treated WT and Plexin B1 KO mice (**Figure 2C**), the mucus secretion and the number of hyperplastic mucous cells per mm of basal lamina were strikingly increased in mice lacking Plexin B1 (**Figure 2D**). The histology photomicrographs shown in **Figures 2E, F** reflect the BALF cell counts and mucus grading/PAS+ cell counts. There was no difference between Plexin B1 KO lungs compared to WT lungs in control mice. However, allergen treatment led to multiple inflammatory infiltrates in both WT and Plexin B1 KO mice. No significant differences in BALF Sema4A levels were detected between PBS controls and OVA experimental groups (data not shown). Plexin B1 deficiency affected BALF cytokines; we observed increased IL-4, IL-5, and TNFα levels (**Figure 2G**) and reduced IL-10 in the BALF of Plexin B1 KO mice compared to WT (**Figure 2G**). The levels of other BALF cytokines and chemokines (IL-25, IL-6, IL-33, eotaxin, and RANTES) did not differ between OVA-treated WT and Plexin B1 KO mice, whereas the levels of TSLP, IFNγ, IL-12, and IL-13 were below the assay’s limit of detection. The effect of Plexin B1 deficiency on the allergic inflammatory response to OVA is similar to the effect of Sema4A deficiency we observed in our earlier studies (23). Namely, Plexin B1 deficiency leads to increases in BALF and lung tissue inflammation, eosinophilia, mucus production, and Th2 cytokines in BALF, but downregulates IL-10. These results demonstrate that Plexin B1 modulates allergic inflammatory responses in mice exposed to two different allergen protocols with some variation in impact between the two models.

### Lower number of Treg cells in lymphoid organs of mice lacking Plexin B1

**Figure 3:**
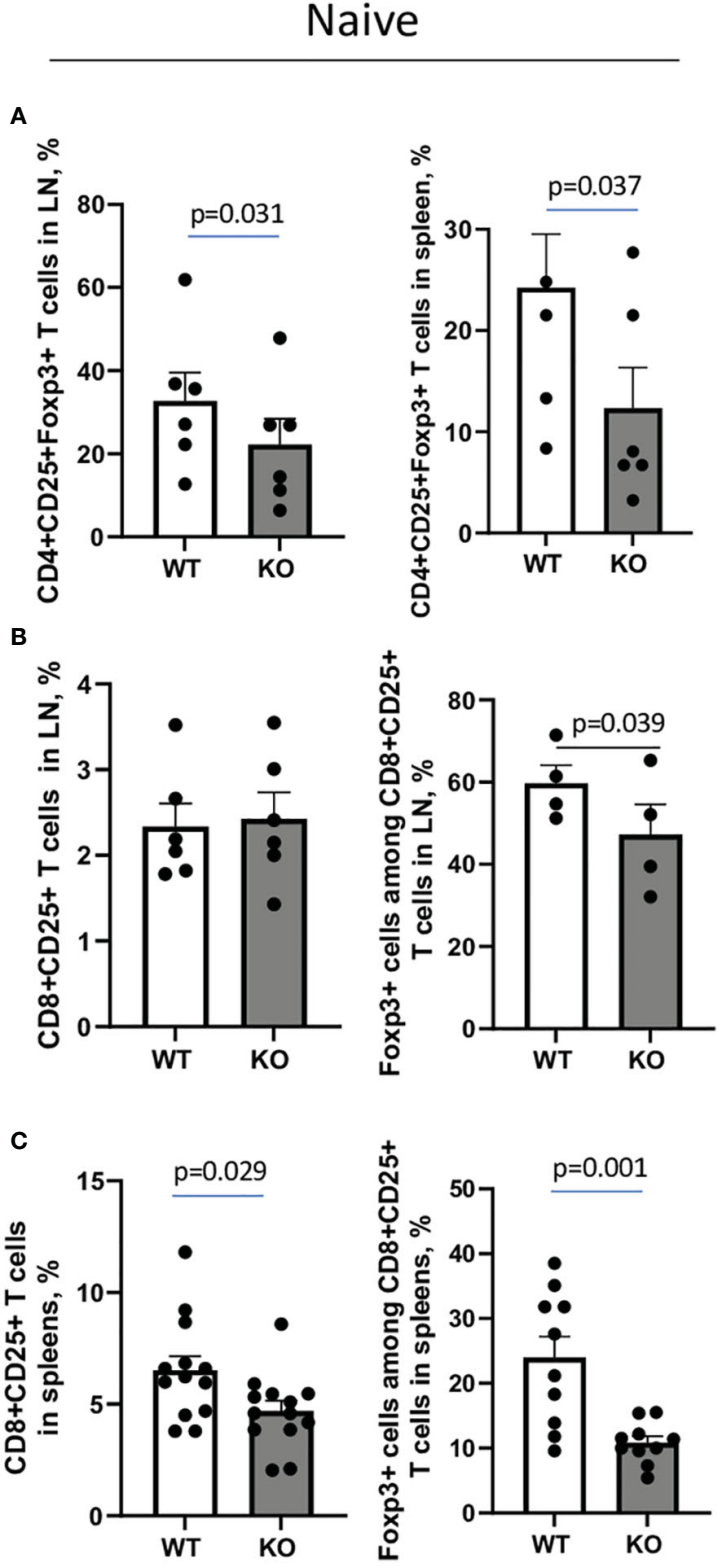
Plexin B1 deficiency results in reduced numbers of Treg cells in lymphoid tissues. Spleen and lymph node cells were isolated from WT and Plexin B1 KO mice and evaluated by flow cytometry using direct fluorochrome-labeled Abs to cell surface and intracellular markers. The data represent combined values from three independent FACS experiments using pooled axillary, popliteal, and mesenteric LN **(A, B)** and two individual spleen samples in each **(A, C)** n=4-6. CD4+CD25+Foxp3+ and CD8+CD25+Foxp3+ cell counts in lymph nodes and spleens of WT and Plexin B1 KO mice in three independent experiments (n=10-12) are shown in corresponding individual dots. The statistical differences between parameters were calculated using the non-parametric paired Mann-Whitney test and one-tailed paired t-test.

The overall percentages of CD3+, CD4+, CD8+, MHCII+B220+ cells, and CD4+CD25+ T cells in lymph nodes (LN) and spleens did not differ between untreated WT and Plexin B1 KO mice (**Supplementary Figure S2**). However, CD4+ and CD8+ Treg cell numbers were affected by Plexin B1 deficiency (**Figures 3A-C**). Among CD4+CD25^high^ lymph node (LN) cells, 32.7% were Foxp3+ in WT mice compared to 22.3% in Plexin B1 KO mice (p=0.031) (**Figure 3A**; **Supplementary Figure S3A**). The difference in CD4+ Treg cell numbers between WT and Plexin B1 KO mice was even more pronounced in spleen cells; there were 24.2% CD4+CD25+Foxp3+ cells in WT spleens compared to 12.3% in Plexin B1 KO spleen cells (**Figure 3A**; **Supplementary Figure S3B**).

We also observed that 2.3% of CD8+ T cells in LN of WT mice expressed CD25, and the majority of these cells, 59.7%, were Foxp3+; these numbers were significantly reduced in Plexin B1 KO mice with 2.4% of CD8+ cells expressing CD25 of which 47.2% were Foxp3+ (**Figure 3B**; **Supplementary Figure S3C**). In spleens, 6.5% of CD8+ cells co-expressed CD25 in WT mice while 4.7% of CD8+ cells co-expressed CD25 in Plexin B1 KO mice (**Figure 3C**; **Supplementary Figure S3D**). While 24% of CD25+CD8+ T cells co-express Foxp3 in WT spleens, only 10.8% did so in spleen cells isolated from Plexin B1 KO mice. Thus, global Plexin B1 deficiency results in reduced CD4+ and CD8+ Treg cell numbers in lymphoid organs at baseline.

### Higher allergic lung inflammatory response in Plexin B1 KO mice is associated with a lower number of Tregs in the lungs

**Figure 4:**
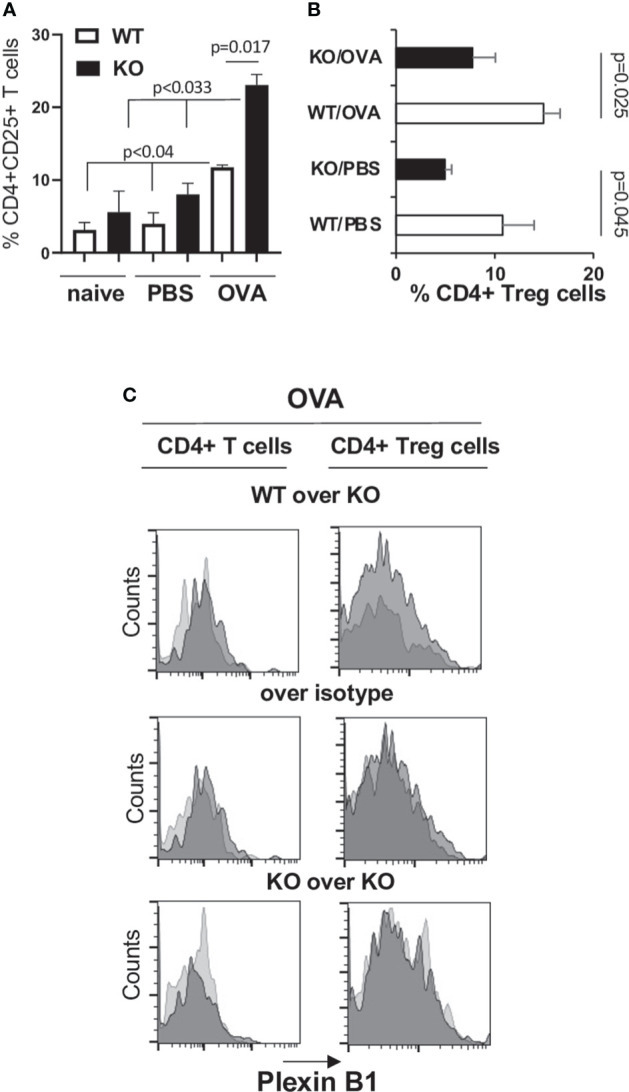
Increased CD4+CD25+ T cell and decreased Treg cell numbers are associated with a heightened allergic response in Plexin B1KO mice. Lungs were excised from OVA-immunized and challenged WT and Plexin B1 KO mice. Lung tissues were digested with collagenase-DNAse and single-cell suspensions were analyzed by FACS for the expression of CD3, CD4, and CD25 markers. **(A)** The numbers of CD25+ cells among CD4+ T cells were assessed by a sequential gating strategy shown in **Supplementary Figure S4A**. The statistical differences between parameters were calculated using the non-parametric paired Mann-Whitney test. **(B)** The percentage of CD4+Foxp3+ Treg cells in PBS- and OVA-treated mice was calculated based on the gating strategy shown in **Supplementary Figure S4B**. Data statistics were calculated using two-tailed Mann-Whitney’s non-parametric test and Wilcoxon’s non-parametric paired test. **(C)** Plexin B1 staining was evaluated on CD4+ T cells and Treg cells as described in Materials and Methods. Histogram overlays were prepared showing Plexin B1 on WT versus KO cells (dark gray *vs* light gray), Plexin B1 on WT cells compared to control Ig (dark *vs* light gray), or Plexin B1 on KO cells compared to control Ig (dark *vs* light gray). The data shown are representative of one out of two independent FACS experiments with two individual lung samples in each.

Since there is a critical role for Tregs in suppression of Th2 response to allergen, we analyzed the numbers of Tregs in the lungs of WT and Plexin B1 KO mice subjected to OVA priming and challenge by multi-color flow cytometry. We observed an increase in CD4+CD25+ T cells in the lungs of OVA-primed Plexin B1 KO mice as compared to WT mice (22% versus 8%, respectively, **Supplementary Figure S4A**; **Figure 4A**). However, there was a significantly lower number of Foxp3+ cells among the CD4+CD25+ in OVA-primed Plexin B1 KO lungs (8%) as compared to WT (15%) counterparts (**Supplementary Figure S4B**; **Figure 4B**). These results suggest that the increased inflammatory response in the lungs of OVA-primed Plexin B1 KO mice is accompanied by increased numbers of activated T cells (CD4+CD25+) but a reduced numbers of Tregs (CD4+CD25+Foxp3+) when compared to WT.

### Treg cells in lungs express low Plexin B1

CD4+ T cells in lungs from OVA-primed WT mice demonstrated low levels of Plexin B1 expression detected by a shift to the right of the anti-Plexin B1 histogram of WT cells when overlayed on stained KO cells or the control Ig stain (**Figure 4C**). Low levels of Plexin B1 were also observed on lung Treg cells in OVA-exposed WT mice (**Figure 4C**). Plexin B1 expression was below detection by Flow cytometry on naïve CD4+ cells and low on naïve CD8+ cells in lymphoid tissues of WT mice (**Supplementary Figure S5A**) consistent with a previous study (39). However, Plexin B1 expression was highly upregulated by *in vitro* ConA stimulation of WT cells but not Plexin B1 KO cells (**Supplementary Figure S5B**). No Plexin B1 staining was detected on cells obtained from Plexin B1 KO mice.

### Plexin B1 deficiency has no effect on Ab response to OVA

**Figure 5:**
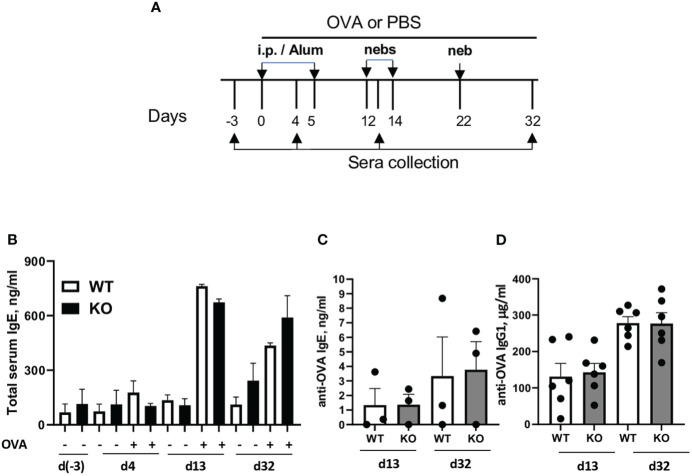
Plexin B1 deficiency has no effect on total and antigen-specific IgE/IgG1 levels. WT and Plexin B1 KO mice were primed and challenged with OVA and sera was collected at various times as described in Materials and Methods **(A)**. **(B)** Total serum IgE and OVA-specific **(C)** IgE and **(D)** IgG1 were measured by ELISA. Data represent one experiment for each assay with sera collected from n=3 mice/group (IgE) and n=6 mice/group (IgG1). *p=0.0006, PBS treated WT *vs* Plexin B1 KO mice, day 32 of experimental protocol, non-parametric unpaired Mann-Whitney test.

Sema4A deficiency had a profound effect on the OVA-specific serum antibodies in allergen challenged mice (23). To investigate if deficiency of the Sema4A receptor, Plexin B1, would mirror this effect, we subjected WT and Plexin B1 KO mice to either PBS or OVA treatments as shown in **Figure 4A**. No differences in the total serum IgE levels between OVA-immunized/challenged WT and Plexin B1 KO mice were found at different time points during the immunization protocol (**Figures 5A, B**). Similarly, OVA-specific IgE and IgG1 concentrations did not differ significantly between WT and Plexin B1 KO mice (**Figures 5C, D**). Therefore, in contrast to our previous findings in mice lacking Sema4A (23), Plexin B1 KO mice did not display upregulation of anti-OVA IgE/IgG1.

### Plexin B1 deficiency affects lung mucin gene expression and Muc5AC secretion

**Figure 6:**
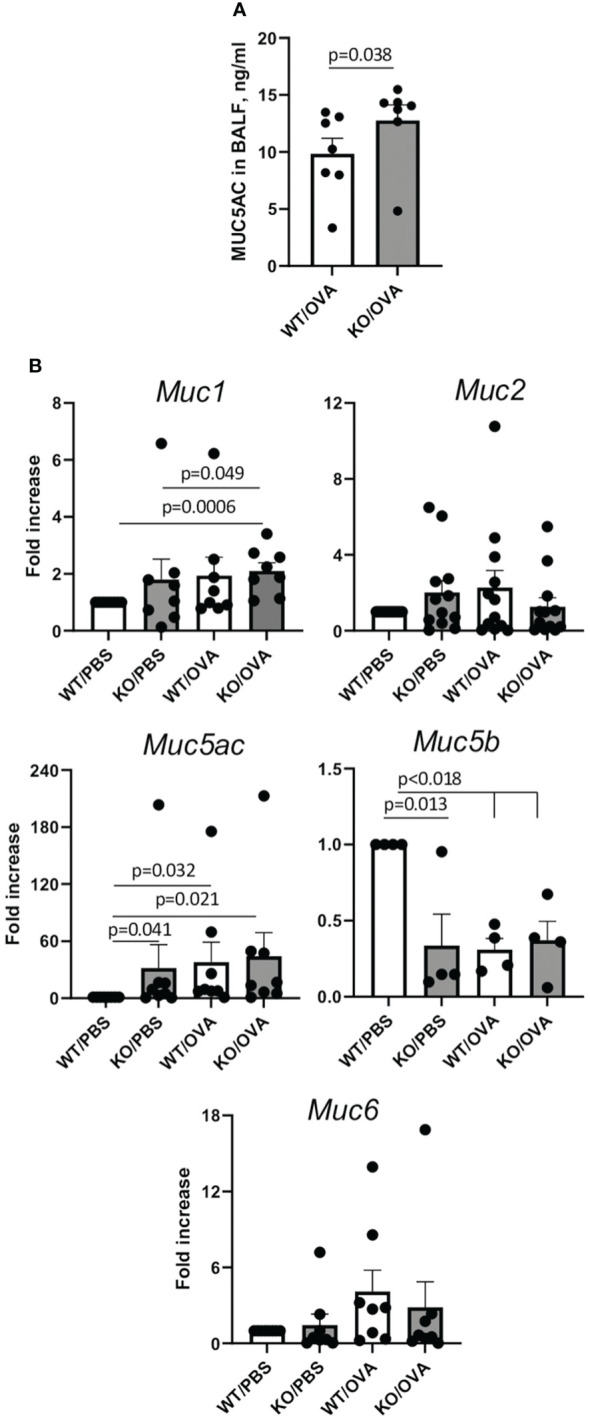
Plexin B1 regulates mucin expression in lungs. BALF was evaluated for MUC5AC by ELISA, n=7 mice/group **(A)**. **(B)** Lung RNA was isolated from the indicated mice and evaluated for mucin gene expression by qRT-PCR as described in Materials and Methods (n=4-8/group). Data shown as mean ± SEM **(A, B)** with data statistics calculated using two-way ANOVA with Tukey’s multiple comparison test.

Mucin dysregulation is one of the damaging and sometimes fatal pathological features of allergic asthma (40, 41). Considering the elevated mucus score observed on PAS-stained lung tissue slides obtained from OVA-treated Plexin B1 KO mouse lungs as compared to their similarly treated WT counterparts (**Figure 2D**), we analyzed Muc5AC levels in BALF by ELISA. We found that Muc5AC was significantly higher in BALF of OVA-treated Plexin B1 KO mice as compared to that in WT mice (**Figure 6A**). We further analyzed the expression of the core mucin mRNA in lung tissue homogenates by RT-qPCR. In WT mice, OVA treatment significantly increased *Muc5ac* mRNA while it reduced *Muc5b* mRNA as compared to PBS control (**Figure 6B**). Interestingly, Plexin B1 KO mice demonstrated increased levels of *Muc1* (**Figure 6B**). The expression levels of elevated *Muc5ac* and decreased *Muc5b* mucins were not further changed in Plexin B1 KO lungs by OVA treatment (**Figure 6B**). Therefore, Plexin B1 deficiency led to Muc1, Muc5ac and Muc5b mucin gene dysregulation in PBS-treated mouse lungs, and to significantly elevated Muc5AC protein levels in BALF with OVA exposure.

### Plexin B1 regulates T cell activation and proliferation in responses to antigen *in vitro*

**Figure 7:**
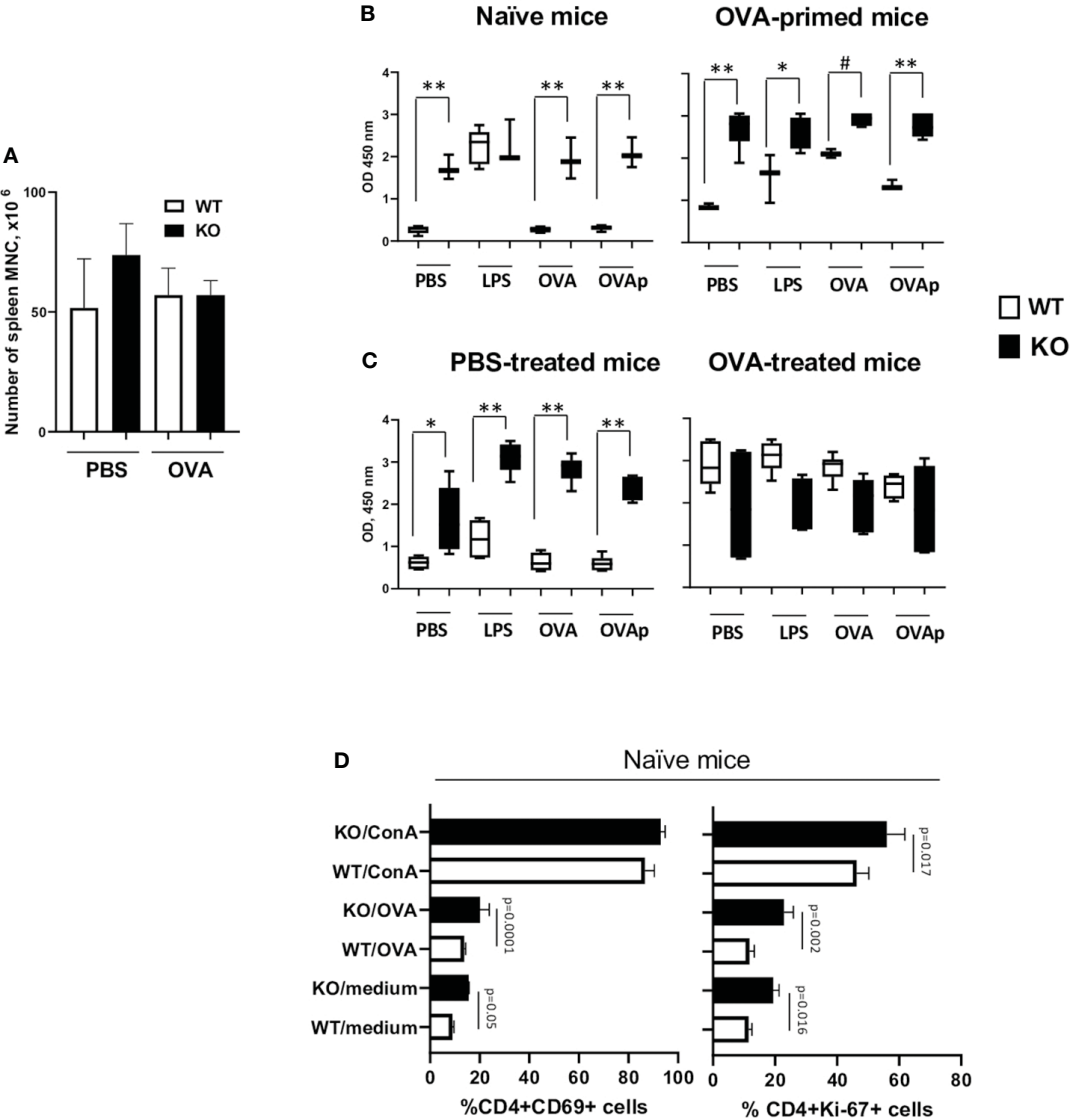
Plexin B1 regulates *in vitro* T cell responses. Spleens were harvested from naïve WT and Plexin B1 KO mice and from OVA-primed counterparts on day 5. **(A)** The total number of isolated mononuclear cells was evaluated. **(B)** Spleen cells were cultured with or without LPS, OVA, and OVA peptide stimulation and evaluated by the WST-1 colorimetric assay. *p=0.005, #p=0.031, **p=0.0001, WT *vs* KO. **(C)** Spleens were harvested mice treated with PBS or OVA as described in **Figure 2** on day 19 (five days after completion of treatment). WST-1 assay was used for colorimetric detection of the formazan dye produced by living cells. *p=0.0017, **p,0.0001, WT *vs* KO. **(D)** Spleen cells from naïve mice were cultured for 36h and analyzed by flow cytometry for CD69 or Ki-67 activation marker expression using gating strategy shown in **Supplementary Figure S6**. Data for triplicate cultures (mean ± SEM) in three *in vitro* experiments are shown (n=3). Data statistics was calculated using two-way ANOVA with Tukey’s multiple comparison test.

We next examined the role of Plexin B1 in T cell activation *in vitro*. We obtained similar numbers of total mononuclear cells (MNC) from spleens of WT and Plexin B1 KO mice (**Figure 7A**). Analysis of *in vitro* responses of spleen cells from naïve or OVA-primed mice using the cell proliferation agent WST1 (23) demonstrated significantly higher numbers of metabolically active Plexin B1 KO cells in culture compared to WT (**Figure 7B**). The metabolic activity of naïve WT cells was low in response to medium, OVA, or OVA-peptide (OVA_323-339_), while it was enhanced in response to LPS. Remarkably, naïve Plexin B1 KO cells demonstrated high levels of metabolic activity when cultured in media alone or stimulated with OVA or OVA-peptide to levels significantly higher than WT cells. A similar pattern of responsiveness was observed when the cells were isolated from PBS-treated mice (**Figure 7C**). WT cells obtained from mice primed 1x with OVA showed basal *in vitro* proliferation that was increased by stimulation with LPS, OVA, or OVA-peptide (**Figure 7B**). Plexin B1 KO cells demonstrated a significantly increased response compared to WT cells in all treatment groups. Interestingly, when spleen cells were prepared from mice exposed to the full OVA/Alum allergic lung inflammation protocol, the *in vitro* recall responses between WT and PlexinB1 KO were not significantly different from each other, however the Plexin B1 KO response tended to be slightly less than WT (**Figure 7C**).

We also examined the proliferative and activation status of cells by Flow cytometric analysis (**Supplementary Figures S6A, B**) for Ki-67 and CD69. We observed that Ki-67 expression was significantly higher in Plexin B1 KO CD4+ cells in the media-, OVA-, and ConA-stimulated cultures as compared to WT cells (**Figure 7D**). The number of CD4+CD69+ cells doubled in Plexin B1-deficient spleen cells cultured with medium alone or stimulated with OVA (**Figure 7D**). Taken together, these results suggest that Plexin B1 plays a role in limiting the proliferative response of naïve splenic T-cells during *in vitro* culture.

### Plexin B1 regulates T cell activation and proliferation *in vivo*

**Figure 8:**
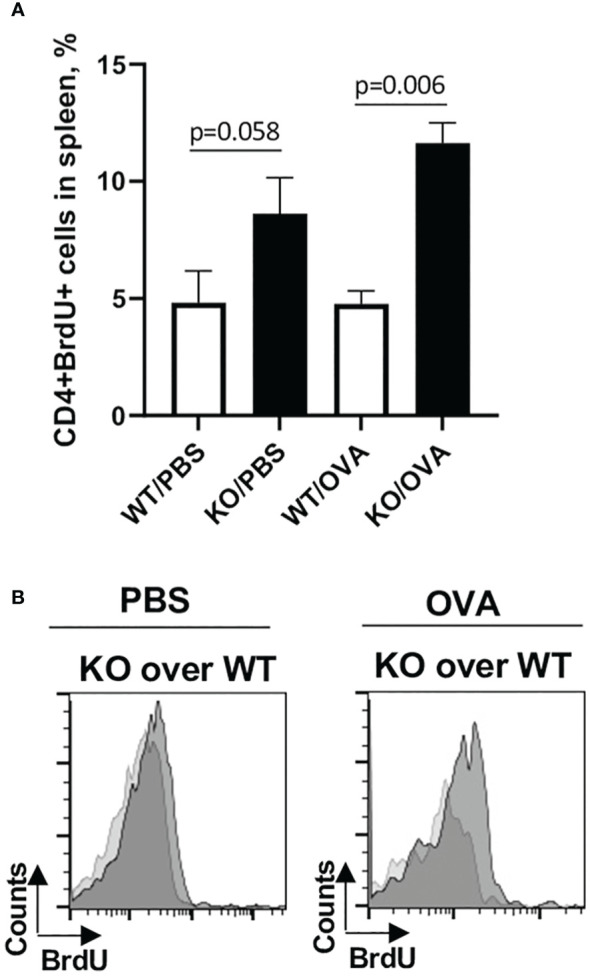
Plexin B1 modulates the *in vivo* CD4+ T cell activation and proliferation. Mice were injected i.p. with either PBS/Alum or OVA/Alum and BrdU as described in Materials and Methods section. Spleens were harvested from mice 24h after last BrdU injection. Spleen MNC were stained with the indicated Abs to cell surface markers and for interchromatin BrdU and analyzed by flow cytometry. The gating strategy used is detailed in **Supplementary Figure S7**. **(A)** The panel shows percentage of CD4+BrdU+ cells in spleens of OVA-treated WT *vs* Plexin B1 KO mice. **(B)** The histograms show BrdU expression levels in Plexin B1KO mice (dark grey) *vs* WT (light grey) mice. Data statistics from two experiments (n=4) was calculated using two-tailed Mann-Whitney’s non-parametric test.

Considering that splenic T cells from Plexin B1 KO mice proliferated more robustly to *in vitro* stimuli as compared to cells obtained from WT spleens, we analyzed *in vivo* T cell activation using the OVA and BrdU treatment of mice followed by interchromatin BrdU staining. PBS/Alum-treated mice served as controls in this study. The number of CD4+BrdU+ cells in PBS/Alum-treated mice was elevated in Plexin B1 KO splenocytes compared to WT. Furthermore, after one OVA/Alum priming there were 2-fold more CD4+BrdU+ cells in Plexin B1 KO mice as compared to WT mice (**Figure 8A**). We also observed the increased relative numbers of CD4+CD69+ cells in spleens of Plexin B1 KO mice (**Supplementary Figure S7**) and a shift toward higher intensity of BrdU expression in these cells as compared to WT mice (**Figure 8B**). Our data suggest that Plexin B1 plays an important role in CD4+ T cell homeostasis at base-line and during priming *in vivo*.

### Plexin B1 KO spleen MNC are Th2-prone

**Figure 9:**
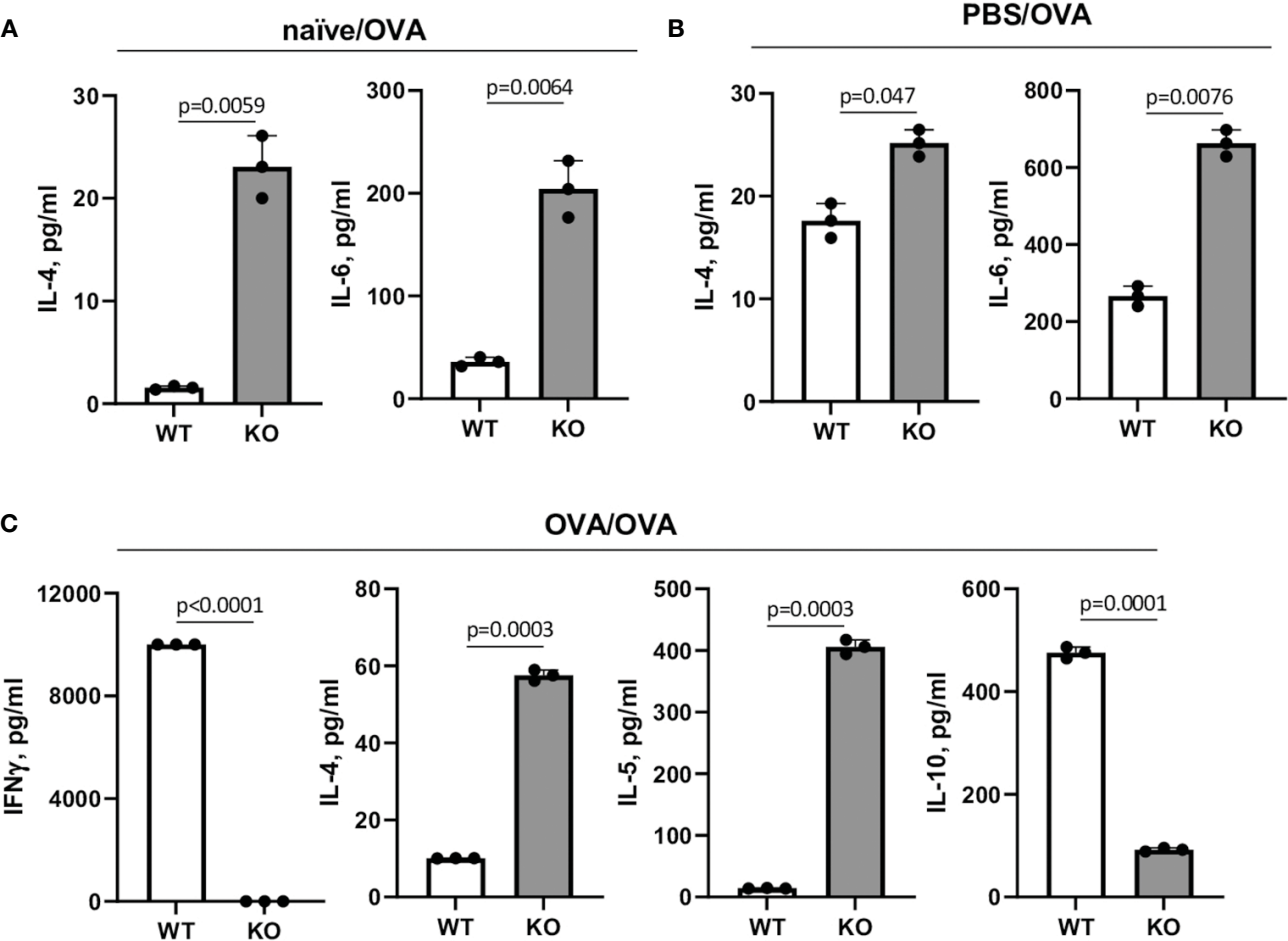
Cytokine levels in spleen MNC cultures. Spleens were harvested from naïve **(A)**, PBS- **(B)** or OVA-injected **(C)** mice five days after last injection. Spleen MNC were cultured in cRPMI in 96-well plate in triplicates for 36h with or without OVA. ConA and LPS stimulations of cells served as positive controls. The amounts of cytokines in cell culture supernatants were measured using Searchlight Proteome Array. Shown are the levels of Th1/Th2 cytokines in different experimental settings which showed the statistically significant differences between WT and KO cells. The levels of IL-13 were below the limit of detection. Data statistics was calculated using two-way ANOVA with Tukey’s multiple comparison test.

Since naïve Plexin B1 KO CD4+ T cells demonstrated enhanced proliferative and activation markers in response to *in vitro* stimuli, we next examined the levels of Th1/Th2 cytokines in the cell culture supernatants. Spleens were obtained from naïve, PBS-treated, and OVA-treated mice. Spleen MNC were cultured *in vitro* with OVA as described in the Materials and Methods section. Cell culture supernatants were collected 36h later and analyzed for Th1/Th2 cytokine content. In the cultures of naïve cells stimulated *in vitro* with OVA, Plexin B1 KO cells already showed measurable IL-4 production as well as IL-6 upregulation as compared to WT cells (**Figure 9A**). For PBS-treated mice, the same cytokines were upregulated in Plexin B1 KO cells (**Figure 9B**). As for *in vivo* OVA-treated and *in vitro* OVA restimulated cells, both IL-4 and IL-5 cytokines were upregulated in Plexin B1 KO cell culture supernatants whereas substantial levels of IFNγ and IL-10 were detected in WT cell cultures (**Figure 9C**). The low level of IL-10 in Plexin B1 KO cell cultures correlates with low Treg cell numbers (**Figures 3B, D**, **9C**, correspondingly). The levels of IL-13 were below (0.64 pg/ml) the assay limits of detection in all samples. Therefore, lymphocytes from Plexin B1 KO mice are already Th2-prone and express significant, detectable levels of IL-4 in cultures. Moreover, immune cells from Ag-primed Plexin B1 KO mice displayed an enhanced Th2 response (IL-4 and IL-5) to *in vitro* Ag restimulation.

## Discussion

We previously showed that Sema4A limited allergic inflammatory responses in part by stabilizing Treg numbers in mice (23). However, Sema4A can utilized at least 3 different counter receptors to transmit signals (10), and thus the counter receptor critical for mediating allergic control has remained unknown. Recently, we reported that Plexin B1 is a critical regulator of human Treg cell stability and function (8). In this study we evaluated whether Plexin B1 acts as an important regulator of mouse Treg cell number and allergic inflammatory responses by developing and analyzing responses in novel Plexin B1 KO mice.

Several other groups previously generated Plexin B1 KO mice, however they were not used to study the immune response or in models of immune mediated diseases such as asthma. One line was generated by inserting a targeting construct into exons 22 and 23 encoding the transmembrane domain of the PlexinB1 gene (42) while another used loxp-mediated deletion of exons 13-16 (43). Our novel Plexin B1 KO mice were generated by Crispr/cas9-mediated deletion of exons 4-16 (**Supplementary Figure S1**). In all cases, Plexin B1 KO mice were found to be viable and fertile without any major developmental defects, somewhat surprising considering the extensive expression of Plexin B1 in different tissues during ontogenesis including the brain and kidney. Plexin B1 was shown to play a redundant role during mouse neuronal development and in tumour angiogenesis (42, 43) likely due to functional overlap with other Plexin family members. Plexin B1 was found to play a role in epithelial branching morphogenesis during kidney organogenesis in one line (26, 43). Korostylev et al. reported that the global Plexin B1 deficiency led to an increase in kidney size as well as ureteric branches at the E13.5 embryonic stage of development likely due to Semaphorin 4D-Plexin B1 interactions (26). Consistent with a role for Plexin B1 in kidney morphogenesis, we observed an increase in kidney size in 11-13 week old male Plexin B1 KO mice (**Supplementary Figure S1B**). In all 3 Plexin B1 mouse lines, histological examination of lung tissues revealed normal morphology of lung epithelium and normal septation of the airways with properly inflated alveoli.

When analyzing responses of these mice to allergens, we observed that the constitutive absence of Plexin B1 led to an upregulation of HDM-induced lung tissue inflammation, mucous cell hyperplasia, and local IL-4 and IL-6 production (**Figure 1**). Plexin B1-deficiency in mice also led to an upregulation of these and additional features of allergic inflammation in the OVA/Alum model including eosinophilia, mucus score and mucous cell hyperplasia, and lung local Th2 cytokine production. Furthermore, there was less IL-10 in BALF of OVA-primed Plexin B1 KO mice consistent with lower numbers of lung Tregs (**Figures 2**, **4**). These results are similar to those observed in Sema4A-deficient mice (23) and thus suggest that Plexin B1may act as an important Sema4A counter receptor for at least some aspects of allergic inflammation.

We found dysregulation of mucin expression in the lungs of allergen-treated Plexin B1 KO lungs as compared WT mice; this was especially evident when analyzing the degree of airway goblet cell metaplasia by density of PAS+ cells per airway (**Figures 1**, **2**). Elevated mucus production is a hallmark of asthma and mucus plugging of the airways is considered a major cause of death in people with asthma (40, 41). Two mucins, MUC5AC and MUC5B, were reported to be the most involved in asthma pathogenesis (44). However, whereas MUC5B is the predominant mucin produced in normal airways in response to external stimuli and does not accumulate pathologically in the airways, MUC5AC production is highly upregulated in response to Th2 cytokines and has a tendency to accumulate and even plug the airways. Murine MUC5B is critical for mucociliary clearance and airway defense. Muc5b-deficient mice do not clear the aspirated materials from the airways, develop chronic bacterial infections, severe inflammation, and airway obstruction. In contrast, MUC5AC-deficient mice have normal mucociliary transport and anti-bacterial defense, but do not develop AHR to allergen challenge (45). Moreover, an inhibition of MUC5AC leads to upregulation of MUC2 and MUC5B expression (44). In our current study, we found increased amounts of MUC5AC secretion in the airways of Plexin B1 KO mice detected in BALF by ELISA (**Figure 6A**) whereas *Muc5B* expression was downregulated after allergen exposure in both mouse lines but also in PBS-treated KO lungs (**Figure 6B**). In addition, *Muc1* transcript levels were upregulated in PBS-treated Plexin B1 KO lungs as compared to WT mice (**Figure 6B**). MUC1 has been reported to play a protective role in allergic asthma as it modulated corticoid resistance in severe asthma, MUC1 levels were downregulated in patients with severe asthma, were inversely correlated with daily doses of inhaled steroids, and MUC1^-/-^ mice were resistant to the anti-inflammatory dexamethasone treatment in OVA model of disease (46). Taken together, the results from the PAS staining and analysis, the Muc5AC ELISA in BALF, and the qRT-PCR for the mucin protein core genes in the lung demonstrate a Plexin B1-dependent mucin dysregulation in mouse lungs.

Interestingly, we did not detect any differences in the antibody responses between OVA-treated WT and Plexin B1 KO mice (**Figures 5B-D**) despite significant differences in allergen-induced lung inflammatory responses (**Figure 2**). This result could possibly be explained by the differential requirements for Th2 effectors and T follicular helper (Tfh) cells important for B cell activation, isotype switching, and resulting allergen-specific Ab secretion [reviewed in (47, 48)]. The report by Kobayashi and associated (49) has shown that natural exposure to allergen induces Th2 and Tfh cells. A conditional deficiency of Bcl6 in CD4+ T cells resulted in a marked decrease of Tfh cell numbers and, as a result, a substantial decrease in anti-allergen IgE antibody levels. However, the Th2 cytokine responses and eosinophilic inflammation in the airways were unaffected by Bcl6 deficiency. Based on these studies, we predict that Plexin B1 may not contribute to Tfh, and that the Sema4A-dependent control of allergen induced antibody responses we reported previously may be mediated by one of its other counter receptors (10, 23).

While studying lymphocyte responses, we obtained evidence for enhanced T-cell activation in Plexin B1 KO mice *in vitro* and *in vivo* (**Figure 7**). There were increased numbers of metabolically active Plexin B1 KO cells cultured *in vitro* at baseline compared to WT, and there were greater numbers of CD4+Ki-67+ and CD4+CD69+ cells *in vitro* even in cells without any previous antigen priming (**Figure 7** D). It is interesting to note that we obtained similar total numbers of spleen cells from naïve WT and KO mice despite the enhanced *in vitro* responses of KO mice, suggesting the presence of other mechanisms *in vivo* that regulate lymphocyte homeostasis.

We also observed enhanced responses in allergen-treated mice. There were greater numbers of CD4+BrDU+ cells *in vivo* after immunization of Plexin B1 KO mice with adjuvant alone (Alum) or OVA/Alum as compared to WT (**Figure 8**). In addition, there were increased numbers of CD4+CD25+ T-cells (Foxp3-) in the lungs of immunized and challenged Plexin B1 KO mice compared to WT (**Figure 4A**). We also found upregulation of Th2 cytokines in BALF from these mice whereas the levels of IL-33 and CCL5 (RANTES) were not affected by Plexin B1 deficiency. Furthermore, there was an upregulation of Th2 cytokines in Plexin B1 KO spleen MNC cultures especially IL-4 and IL-6 in naïve and PBS-primed cells, and IL-4 and IL-5 in OVA-primed cells whereas IL-10 was greatly downregulated and IFNγ undetected as compared to similarly exposed WT cells (**Figures 9A-C**).

These enhanced parameters of T-cell activation were accompanied by a decrease of Treg cell numbers in lymphoid tissues (**Figure 3**) and lungs (**Figure 4**) in Plexin B1 KO mice compared to WT, which we believe explains in part the upregulated inflammatory lung response to allergen. We observed lower numbers of Treg cells in the periphery of mice lacking Plexin B1 consistent with the ability of Sema4A to stabilize human Tregs through Plexin B1 *in vitro* (8). Our data indicate that Plexin B1 is critically involved in allergic asthma and plays a suppressor role in this disease in part by promoting and stabilizing Treg cells. However, the mechanism by which Plexin B1 may stabilize Tregs in the mouse is unclear.

We reported previously that human peripheral CD4+ T cells express Plexin B1 on the cell surface (8). In this study, we observed that naïve mouse CD4+ T-cells express little to no detectable surface Plexin B1 while naïve CD8+ cells express low levels at baseline (**Supplementary Figure S5**) consistent with a previous report (39). However, CD4+ T cells and Treg cells in the lung tissues of OVA-treated mice demonstrated low Plexin B1 expression (**Figure 4C**). In addition, the *in vitro* polyclonal stimulation of T cells led to significant upregulation of Plexin B1 expression levels on both, CD4+ and CD8+ cells (**Supplementary Figure S5B**) suggesting the Plexin B1 expression is induced during activation. A recent study by Naito et al. (39) also demonstrated low levels of Plexin B1 expression on tumor-infiltrating CD4+ and CD8+ T cells. They showed that the tumor cell-derived Sema4A engagement of Plexin B1 on CD8+ T cells increased the effector function of CD8+ T cells and improved the anti-tumor efficacy of PD-1 blocking Ab. Thus, it is possible the mechanism by which global Plexin B1 deficiency modulates T-cell responses and Treg numbers is via a direct T-cell intrinsic pathway.

It is also possible that the effect of Plexin B1 deficiency on Treg numbers is through an alternate, indirect mechanism. Previous reports showed the importance of local IL-33 levels for tissue-resident Treg cells to migrate and expand (50–53). We did not find any difference in BALF IL-33 levels between PBS- or allergen-treated WT and Plexin B1 KO mice (data not shown) whereas Treg cell numbers differ significantly (**Figure 4**). However, IL-33 is known as an important mediator of Th2 immune response by enhancing the function of OX-40L (54). Bronchial epithelial cells also express low levels of Plexin B1 at baseline and strongly upregulate its expression with OVA exposure (29), suggesting that epithelial cell Plexin B1 may regulate epithelial cell responses including goblet cell hyperplasia and cytokine production. Furthermore, using a flow cytometry approach we found Plexin B1 expression on mouse lung DC (29) whereas other reports showed its expression on human follicular DC and *in vitro* activated T cells (55, 56). Therefore, future studies will be needed to clarify the precise mechanisms by which Plexin B1 modulates specific allergic responses in sufficient detail to establish it as an essential immunomodulator.

## Data availability statement

The raw data supporting the conclusions of this article will be made available by the authors, without undue reservation.

## Ethics statement

The animal study was approved by University of Maryland School of Medicine Animal Care and Use Committee. The study was conducted in accordance with the local legislation and institutional requirements.

## Author contributions

SC: Conceptualization, Data curation, Formal analysis, Investigation, Methodology, Writing – original draft, Writing – review & editing. HG: Formal analysis, Investigation, Writing – review & editing. RF: Formal analysis, Investigation, Writing – review & editing. AK: Conceptualization, Data curation, Funding acquisition, Project administration, Resources, Supervision, Validation, Writing – review & editing.
