# Supplementary material for: Plexin B1 controls Treg numbers, limits allergic airway inflammation, and regulates mucins
Source: Front Immunol. 2024 Jan 8;14:1297354. doi: 10.3389/fimmu.2023.1297354 (PMC10801081; doi:10.3389/fimmu.2023.1297354)
Supplement: Supplementary file 1 [file DataSheet_1.pdf]

Supplemental Material

Plexin B1 controls Treg numbers, limits allergic airway inflammation, and regulates mucins

Svetlana P. Chapoval, Hongjuan Gao, Rachel Fanaroff, and Achsah D. Keegan*

*** Correspondence:** Achsah D. Keegan: akeegan@som.umaryland.edu

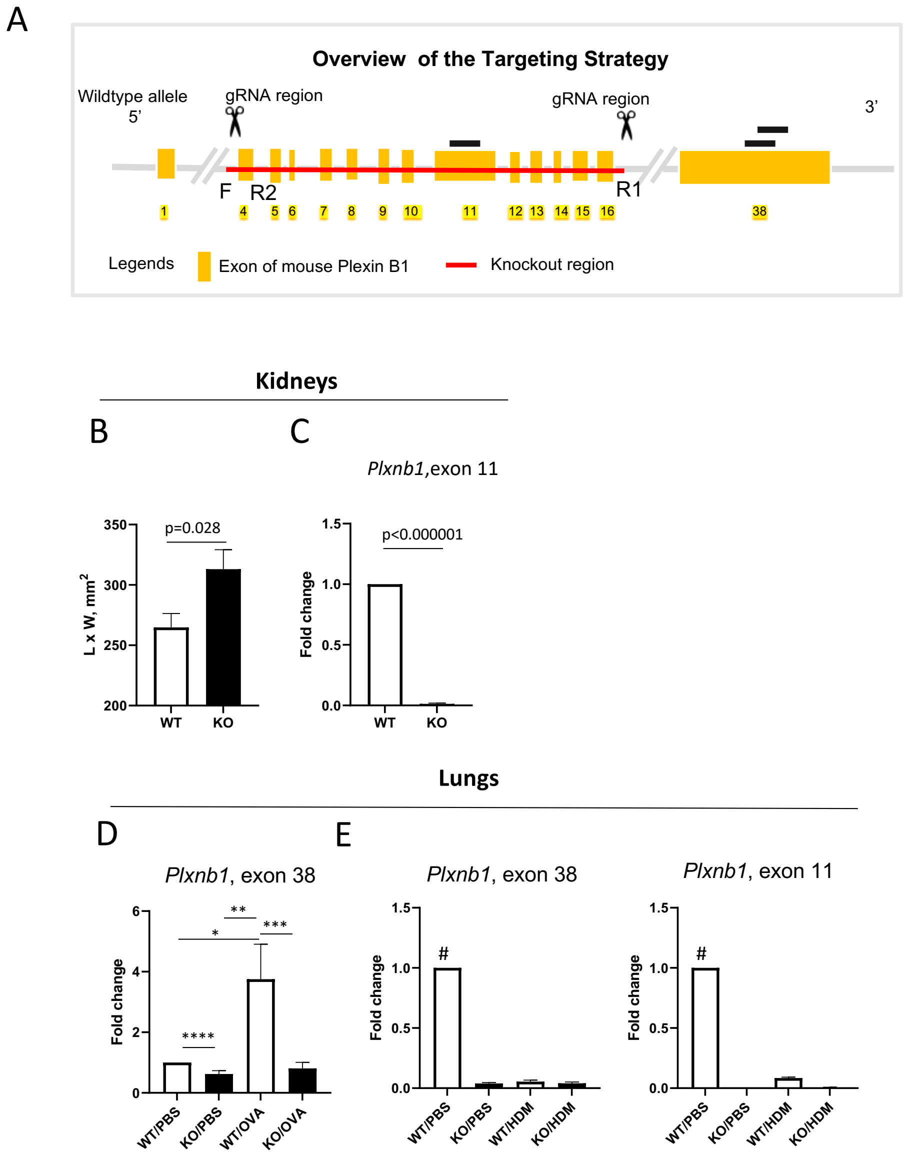

<u>Supplemental Figure S1. Schematic presentation of Plexin B1 KO mouse generation and supportive data of global Plexin B1 deficiency.</u> (A) The knockout region in the PLXNB1 gene including Exons 4-16 is shown. Black lines mark the regions of DNA amplification in qPCR with primers sets used for exons 11 and 38 as shown in Supplemental Table 1. (B) Kidneys were dissected from male WT and KO mice at 13 weeks of age. The kidney size was measured and expressed as a mean of the area (L x W). Data are shown as mean ± SEM for 4 mice/group. (C) Expression of *Plxnb1* mRNA in kidney isolated from WT and Plexin B1 KO mice was evaluated by qRT-PCR as described in Materials and Methods using primer pairs to target sequences located in Exon 11 (n=4/group). Mice were treated with either OVA (D) or HDM (E) using protocols detailed in Materials and Methods (n=5/group). Lung RNA was obtained and evaluated for *Plxnb1* mRNA by qRT-PCR using primer pairs located in Exon 11 and Exon 38 as indicated. *p=0.025, **p=0.012, ***p=0.018, two-way ANOVA with multiple comparisons and ****p=0.028, two-tailed nonparametric Mann-Whitney test. #WT/PBS vs all other experimental groups, p<0.0001. *Plxnb1*, exon's 11 and 38 gene expression for KO/PBS and KO/HDM is not significantly different.

Supplemental Table 1. qPCR primers

Gene	Sense/Antisense	Primer sequences (from 5' to 3')	Reference
Muc1	S<br>AS	CCC CAG TTG TCT GTT GGG TC<br>GGA TTC TAC CAC CAC GGA GCC	36
Muc2	S<br>AS	GCT GAC GAC TGG TTG GTG AAT G<br>GAT GAG GTG GCA GAC AGG AGA C	37
Muc5ac	S<br>AS	GGA CCA ACT GGT TTG ACA CTG AC<br>CCT CAT AGT TGA GGC ACA TCC CAG	36
Muc5b	S<br>AS	GTG AGG AGG ACT CCT GTC AAG T<br>CCT CGC AGA AGG TGA TGT TG	38
Muc6	S<br>AS	TGG TCG AAG TAC TCA TTC TGG<br>GTG GCT TGT GTG GCA ACG CC	37
Plxnb1, exon 38	S<br>AS	GGT CCA CCT TGA TTG CAG GTC<br>CAC TGC CTG GAA TCG CCT TTA	17
Plxnb1, exon 38	S<br>AS	CCT TAA AGA TGG GGG ACA GA<br>CAC CAC CCC TCT GCT AAG AT	35
Plxnb1, exon 11	S<br>AS	CAT GAG AAG CCC CTT CCT CC<br>AAG AGG TCC TCA GGT GTG GT	

S, sense; AS, antisense

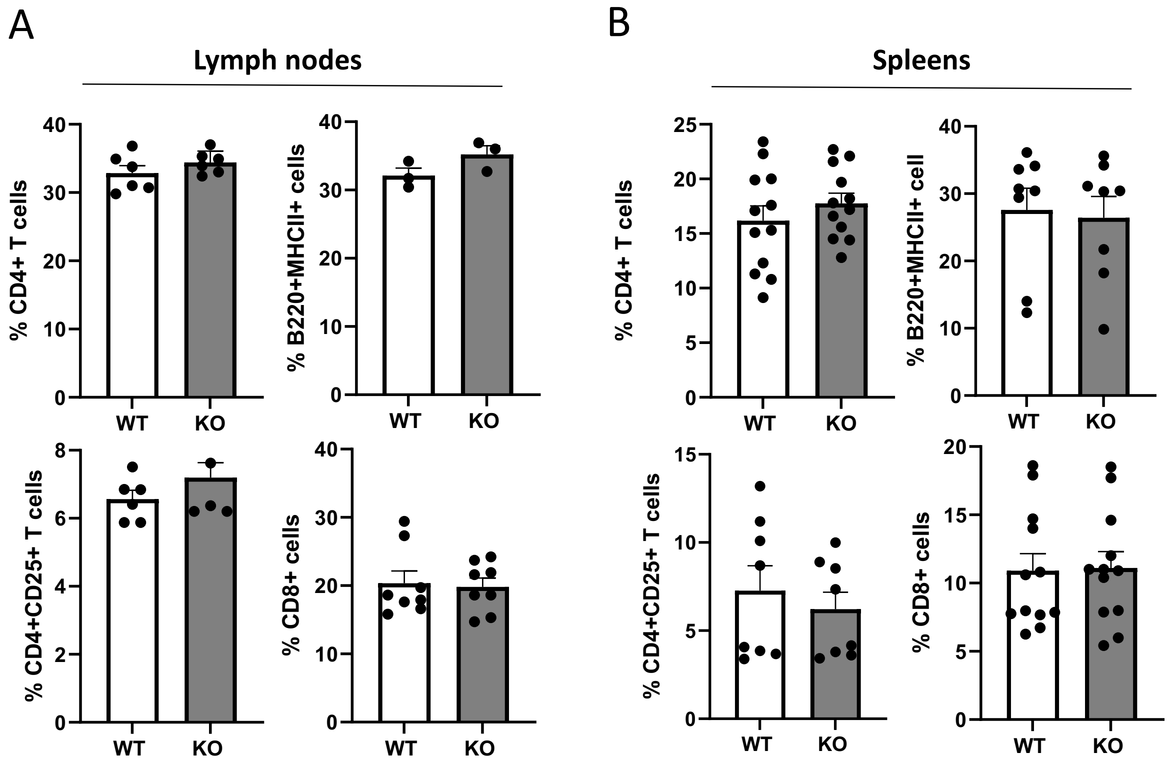

<u>Supplemental Figure S2. Plexin B1 deficiency has no effect on the relative numbers of immune cells in lymphoid tissues.</u> Spleen and lymph node immune cell composition in C57BL/6 and Plexin B1 KO mice was assessed by flow cytometry using direct fluorochrome-labeled Abs to cell surface and intracellular markers. The relative numbers of CD4+ T cells and CD4+CD25+ T cells were not changed in lymph nodes (A) and spleens (B) of mice lacking Plexin B1 as compared to WT counterparts. The data represent combined values from three independent FACS experiments with one combination of axillary, popliteal, and mesenteric LN and two individual spleen samples in each (n=6-12). The statistical differences between parameters were calculated using the non-parametric paired Mann-Whitney test.

Figure S3, A-B

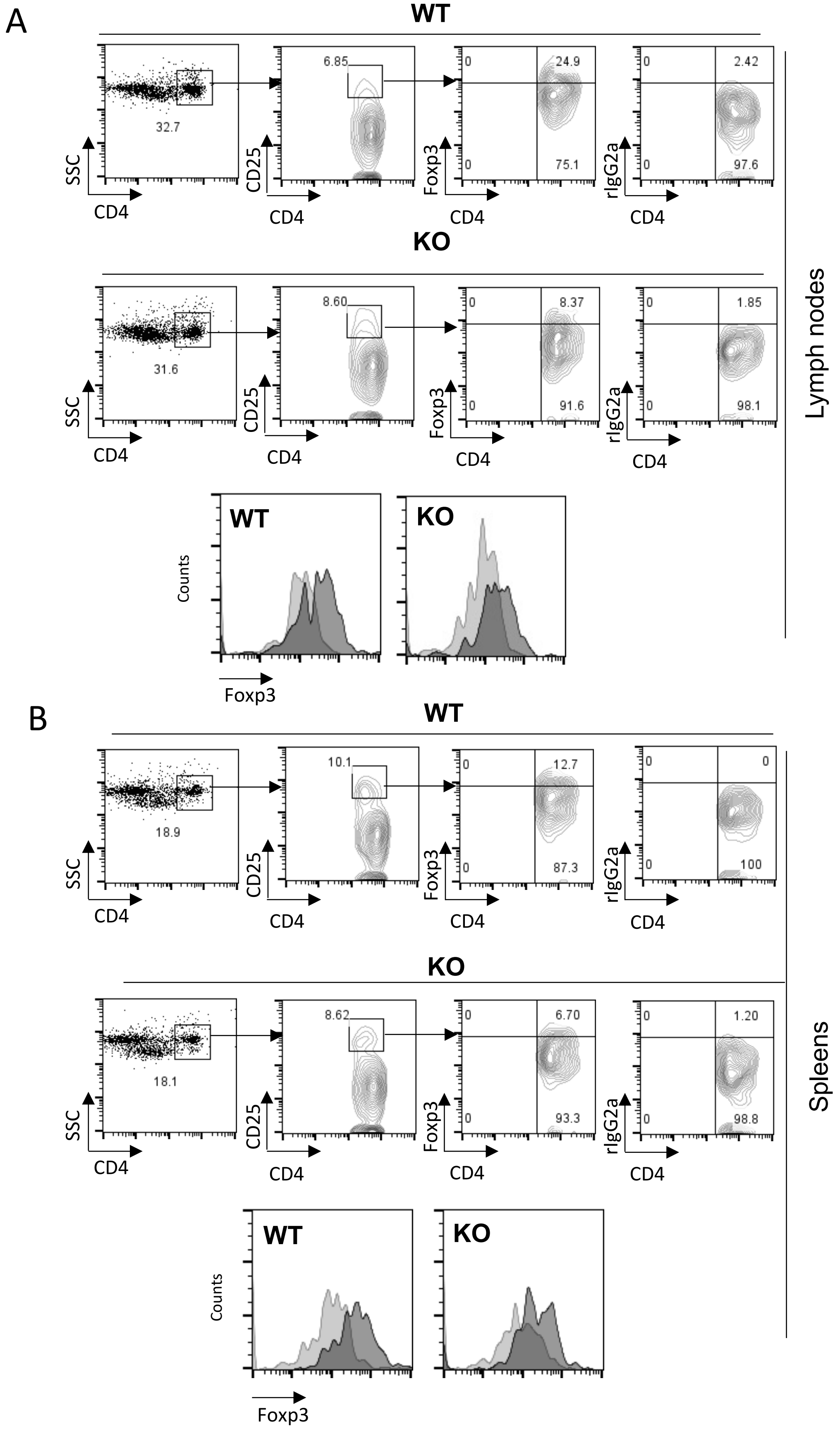
A
WT
KO
B
WT
KO
Lymph nodes
Spleens
SSC
CD4
CD25
Foxp3
rIgG2a
Counts
32.7
6.85
24.9
75.1
2.42
97.6
31.6
8.60
8.37
91.6
1.85
98.1
18.9
10.1
12.7
87.3
100
18.1
8.62
6.70
93.3
1.20
98.8

Figure S3, C-D

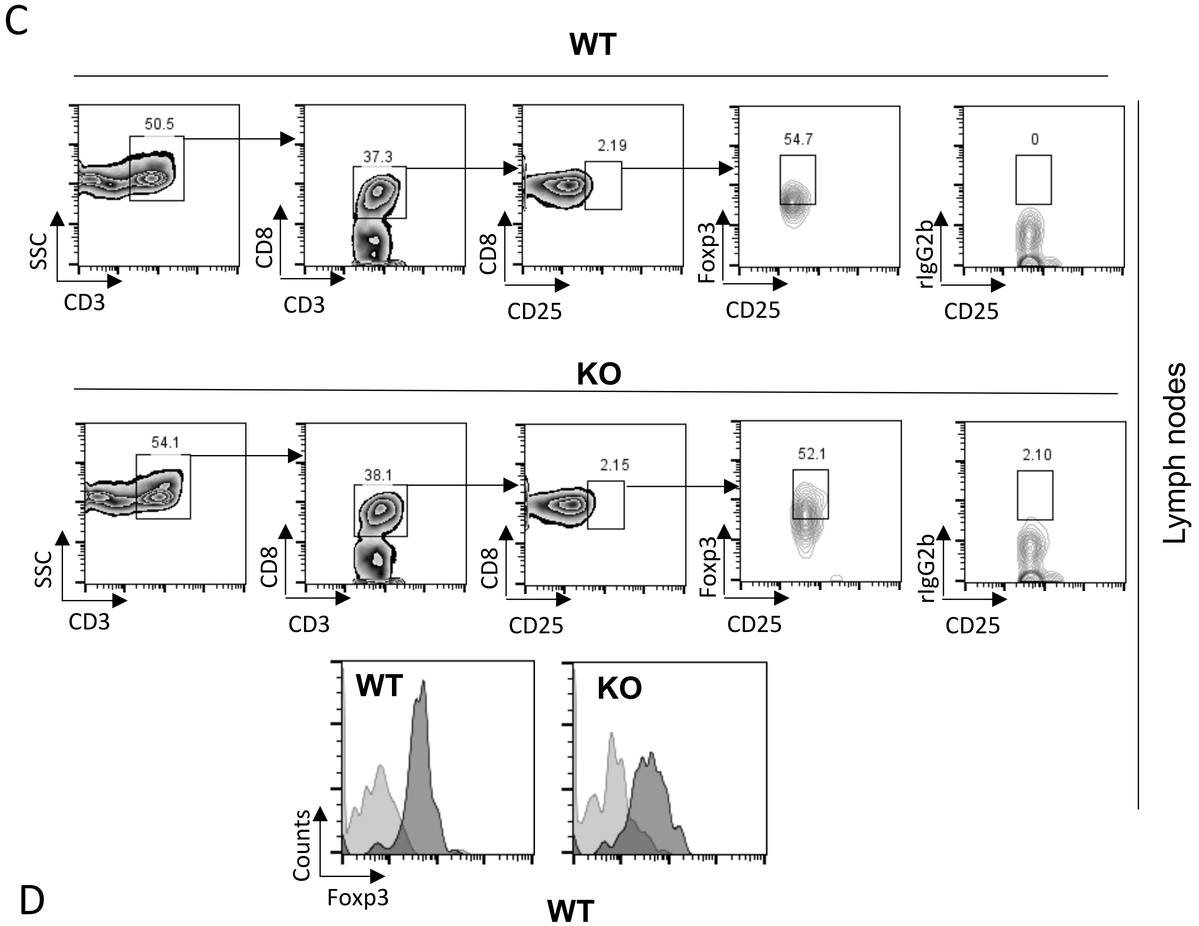
C
WT
KO
50.5
37.3
2.19
54.7
0
54.1
38.1
2.15
52.1
2.10
SSC
CD3
CD8
CD25
Foxp3
rIgG2b
Lymph nodes
Counts
Foxp3

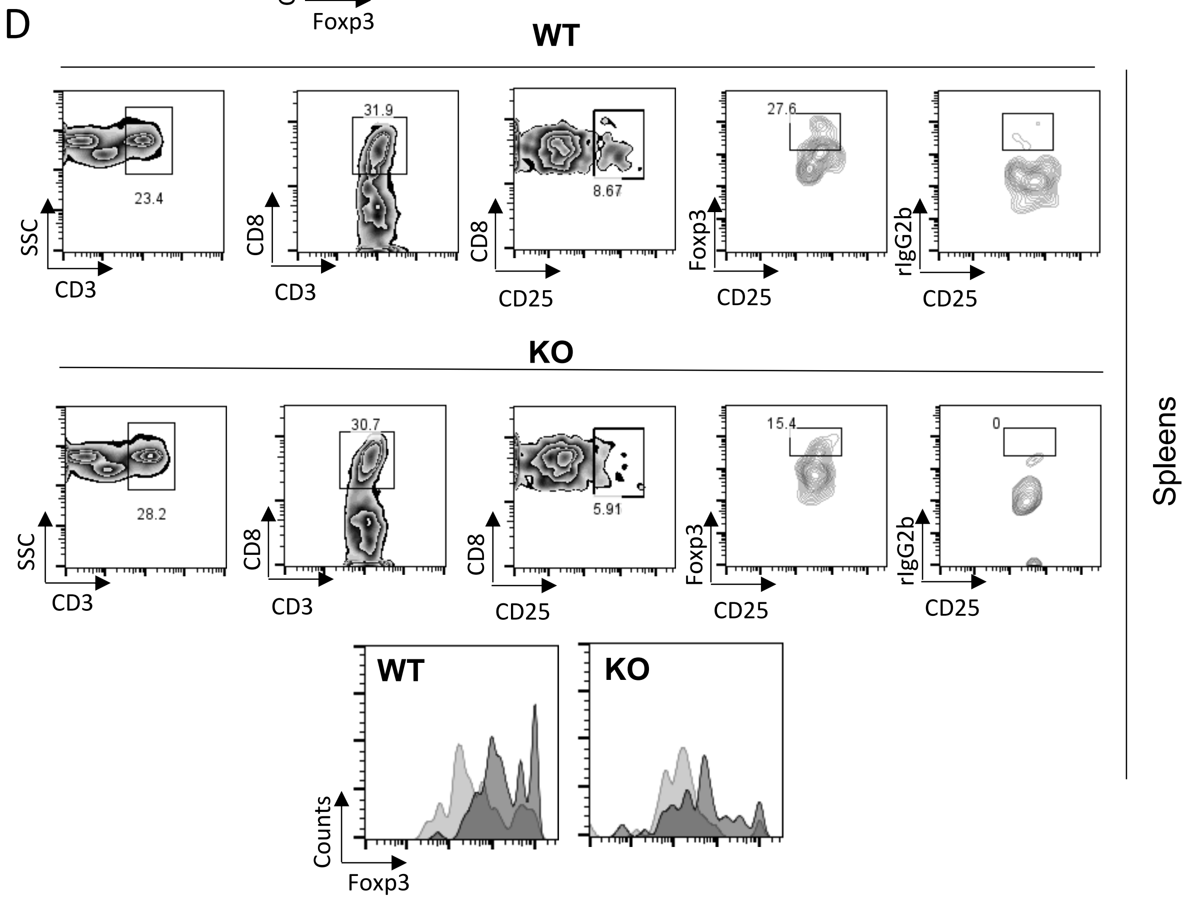
D
WT
KO
23.4
31.9
8.67
27.6
28.2
30.7
5.91
15.4
0
SSC
CD3
CD8
CD25
Foxp3
rIgG2b
Spleens
Counts
Foxp3

Supplemental Figure S3. Gating strategy used in flow cytometry for Treg cell determination in mouse spleens and lymph nodes. (A, B) The right panel shows the sequential gating strategy for Treg cell determination where LN and spleen MNC were gated on CD4+ cells on dot plots and CD4+CD25^{high} cells were selected on contour plots for the evaluation of Foxp3 expression. (C, D) The sequential gating strategy for CD8+CD25+Foxp3+ T cell determination is shown for lymph node (C) and spleen (D). The histograms show the fluorescent intensity of Foxp3 expression (dark grey) on CD4+CD25+ T cells (A, B) and CD8+CD25+ T cells (C, D) over isotype control Ab (light grey) staining.

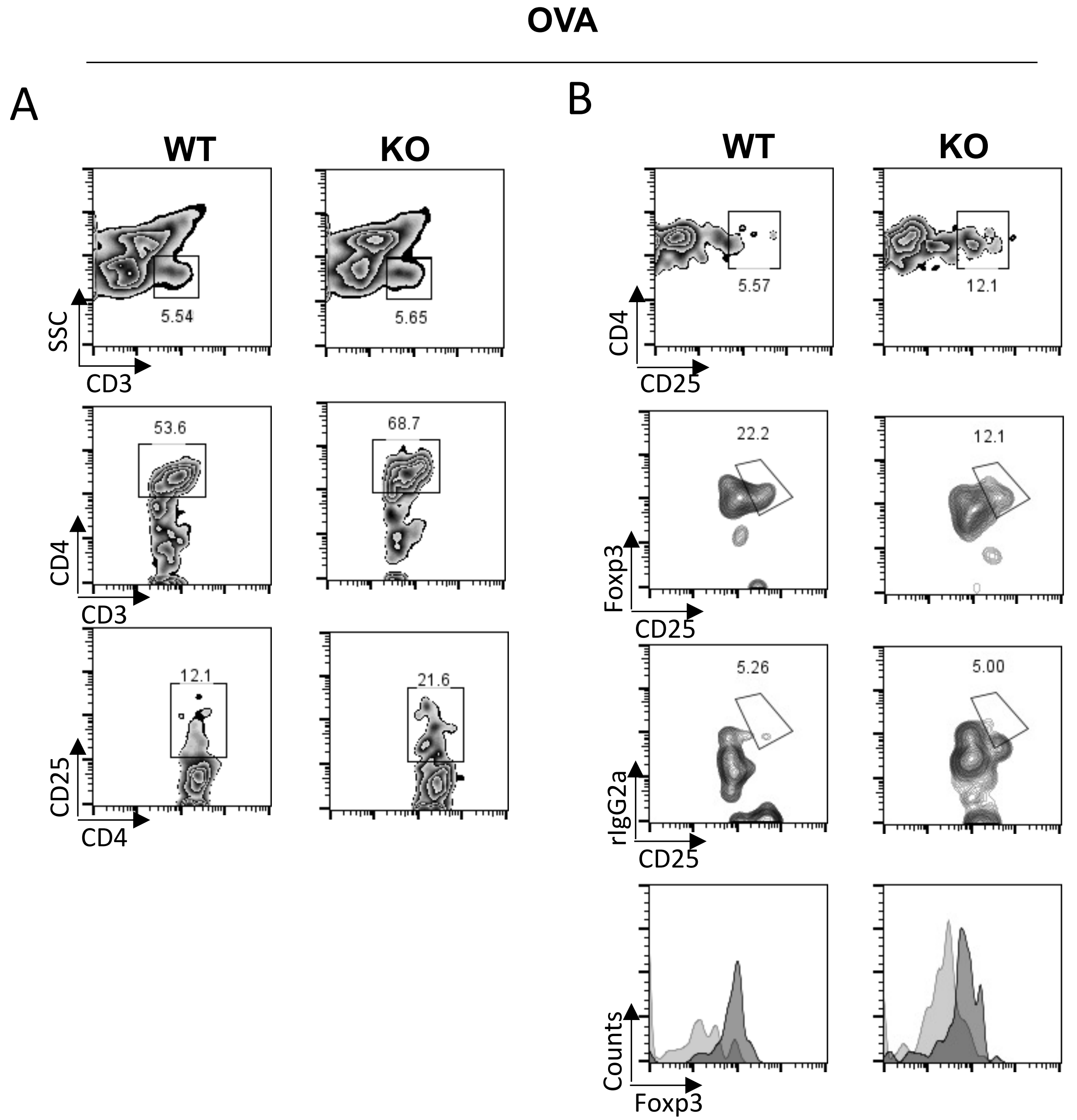

Supplemental Figure S4. Gating strategy used in flow cytometry for Treg cell determination in mouse lungs. Lung tissues were digested with collagenase-DNAse and single-cell suspensions were analyzed by FACS for the expression of CD3, CD4, and CD25 markers (A). (B) Gated CD4 + T cells were further analyzed for high CD25 marker expression, the expression of Foxp3, and the relative number of CD4 + CD25^{high}Foxp3 + cells was ascertained in comparison with a control rat IgG2a protein stain. The histograms show the fluorescent intensity of Foxp3 expression (dark grey) on CD4+CD25+ T cells over isotype control Ab (light grey) staining.

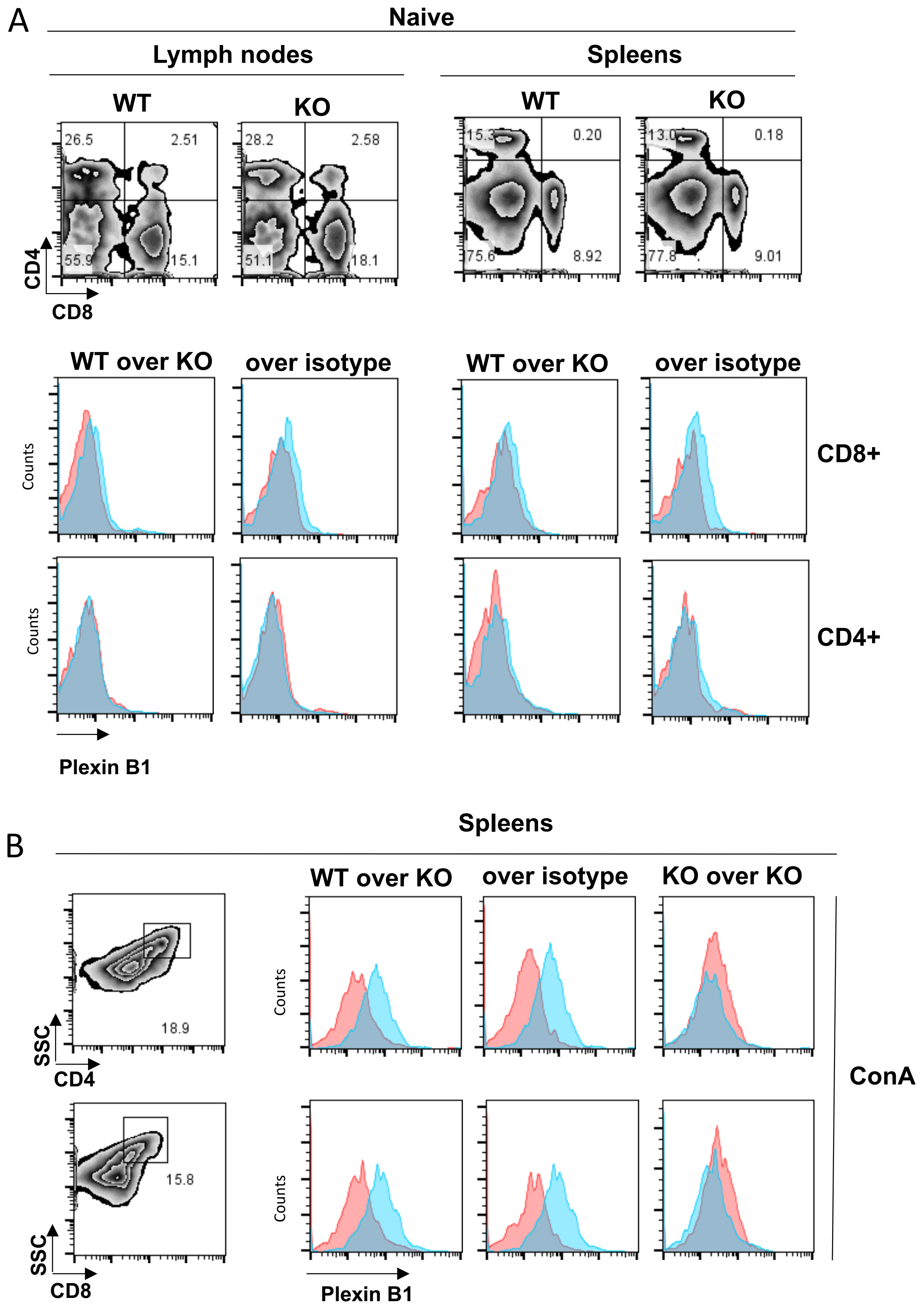

<u>Supplemental Figure S5. Plexin B1 expression is induced on activated CD4+ and CD8+ cells.</u> (A) Spleens and lymph nodes cells were isolated from naïve WT and Plexin B1 KO mice stained anti-Plexin B1 or control Ig with a panel of Abs to T cell markers. Plexin B1 expression was analyzed on CD4+ and CD8+ cells and histogram overlays for anti-Plexin B1 Ab over anti-mouse IgG Ab for WT and KO cells, and Ab to Plexin B1 WT over KO staining are shown. (B) Spleen cells from naïve WT and Plexin B1 KO mice were cultured *in vitro* with ConA. The cells were stained with antibodies to CD4, CD8, and Plexin B1. as indicated. Histogram overlays were prepared showing Plexin B1 on WT versus KO cells (blue vs pink), Plexin B1 on WT cells versus control Ig (blue vs pink), or Plexin B1 on KO cells versus control Ig (blue vs pink). The data are representative of one out of three independent FACS experiments with two individual spleen samples in each.

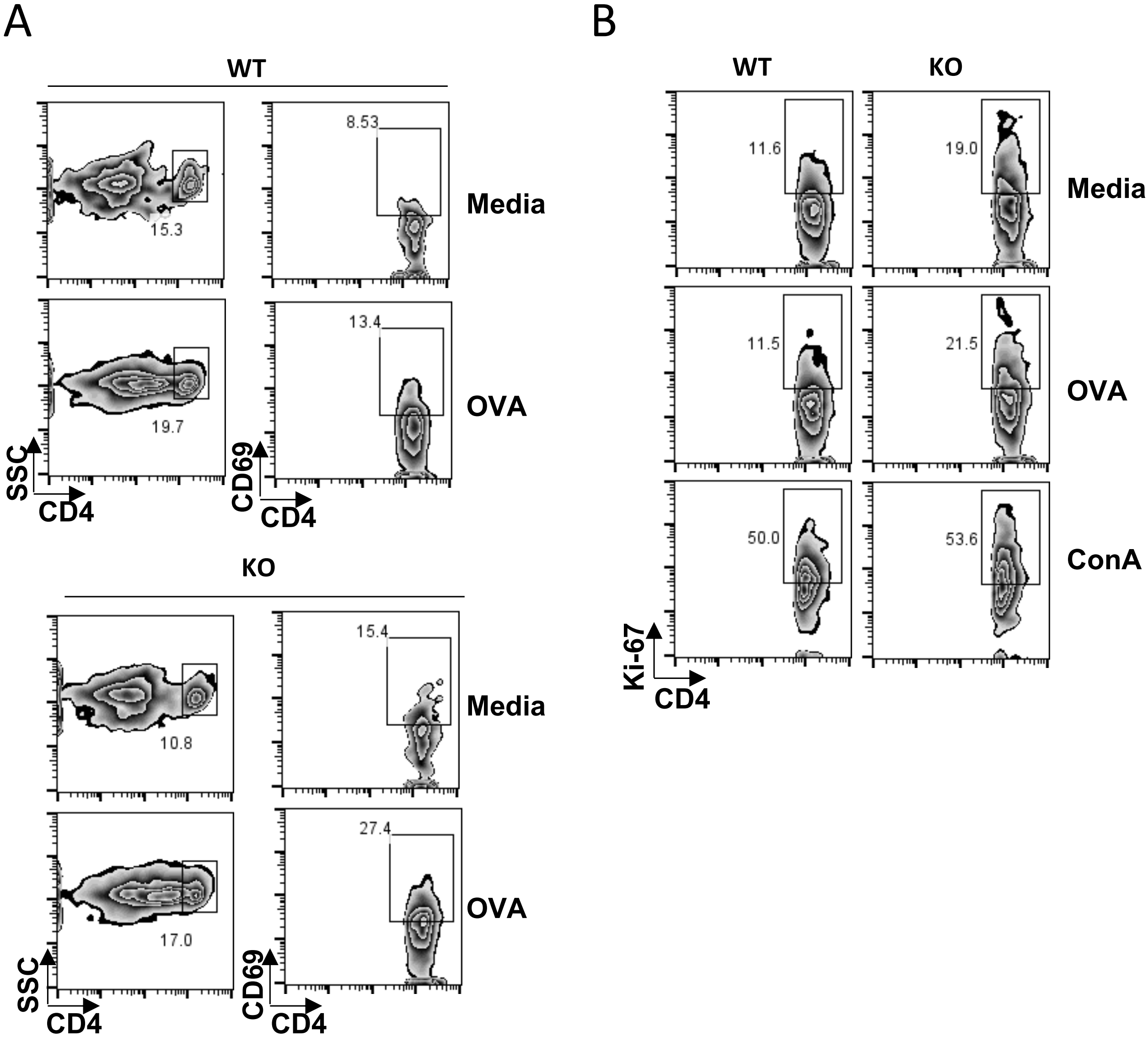

<u>Supplemental Figure S6. Gating strategy used in flow cytometry for the expression of activation markers on spleen MNC.</u> Spleens were obtained from naïve C57BL/6 and Plexin B1 KO mice and processed for single cell suspension. Spleen cells were cultured for 36h with or without OVA, ConA, and analyzed by flow cytometry by first gating on live cells using a dead cell exclusion marker followed by gating on CD4+ cells, and then on CD69 (A) or Ki-67 (B) expression.

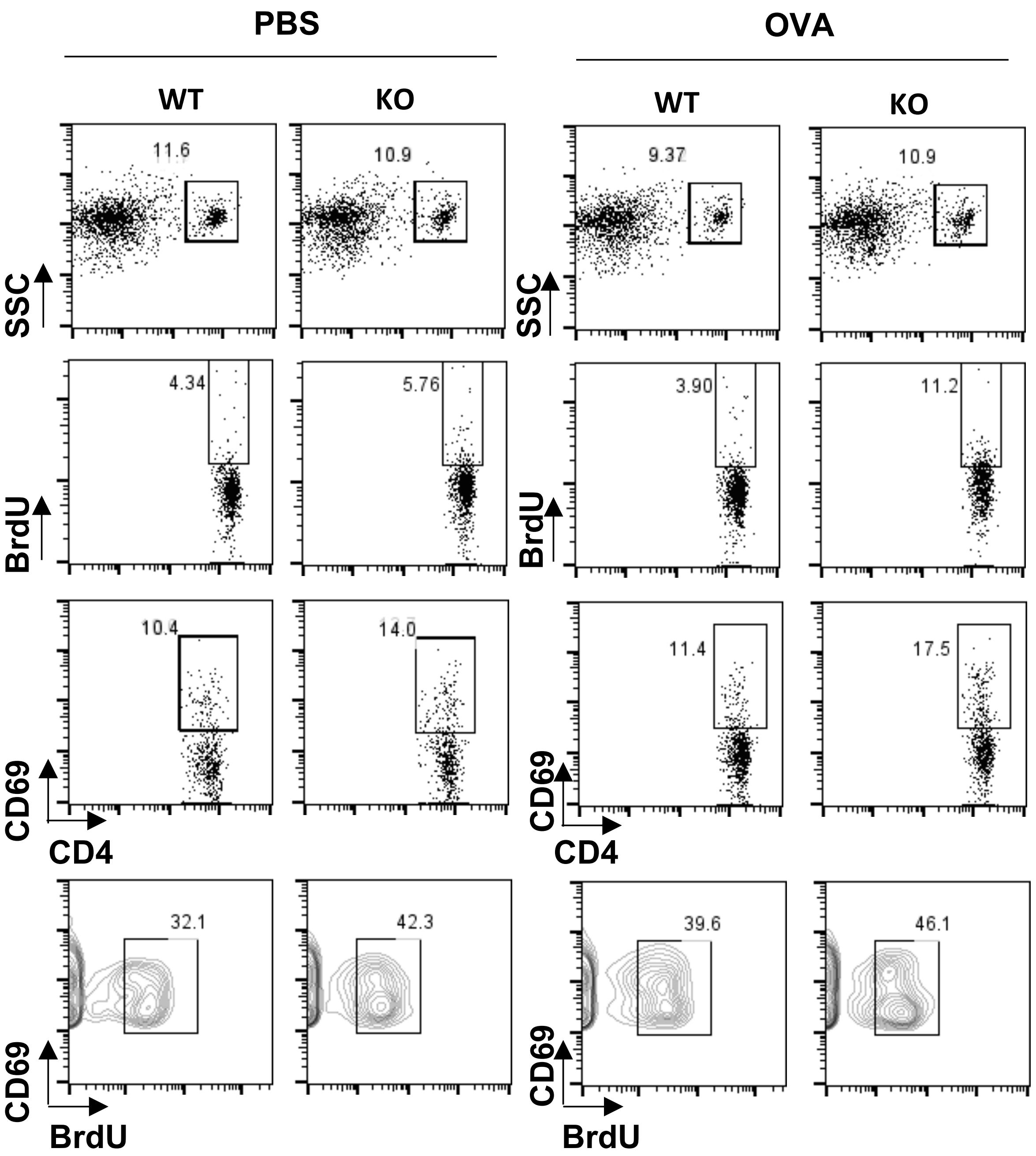

<u>Supplemental Figure S7. Gating strategy used in flow cytometry for the *in vivo* spleen MNC proliferation assay.</u> Spleen MNC were stained with the indicated Abs to cell surface markers and for interchromatin BrdU and analyzed by flow cytometry. The dot plot data shown are from one out of two representative *in vivo* experiments with n=2/group in each.
