# Supplementary material for: Plexin B1 controls Treg numbers, limits allergic airway inflammation, and regulates mucins
Source: Front Immunol. 2024 Jan 8;14:1297354. doi: 10.3389/fimmu.2023.1297354 (PMC10801081; doi:10.3389/fimmu.2023.1297354)
Supplement: Supplementary file 2 [file Presentation_1.pptx]

## Slide 1
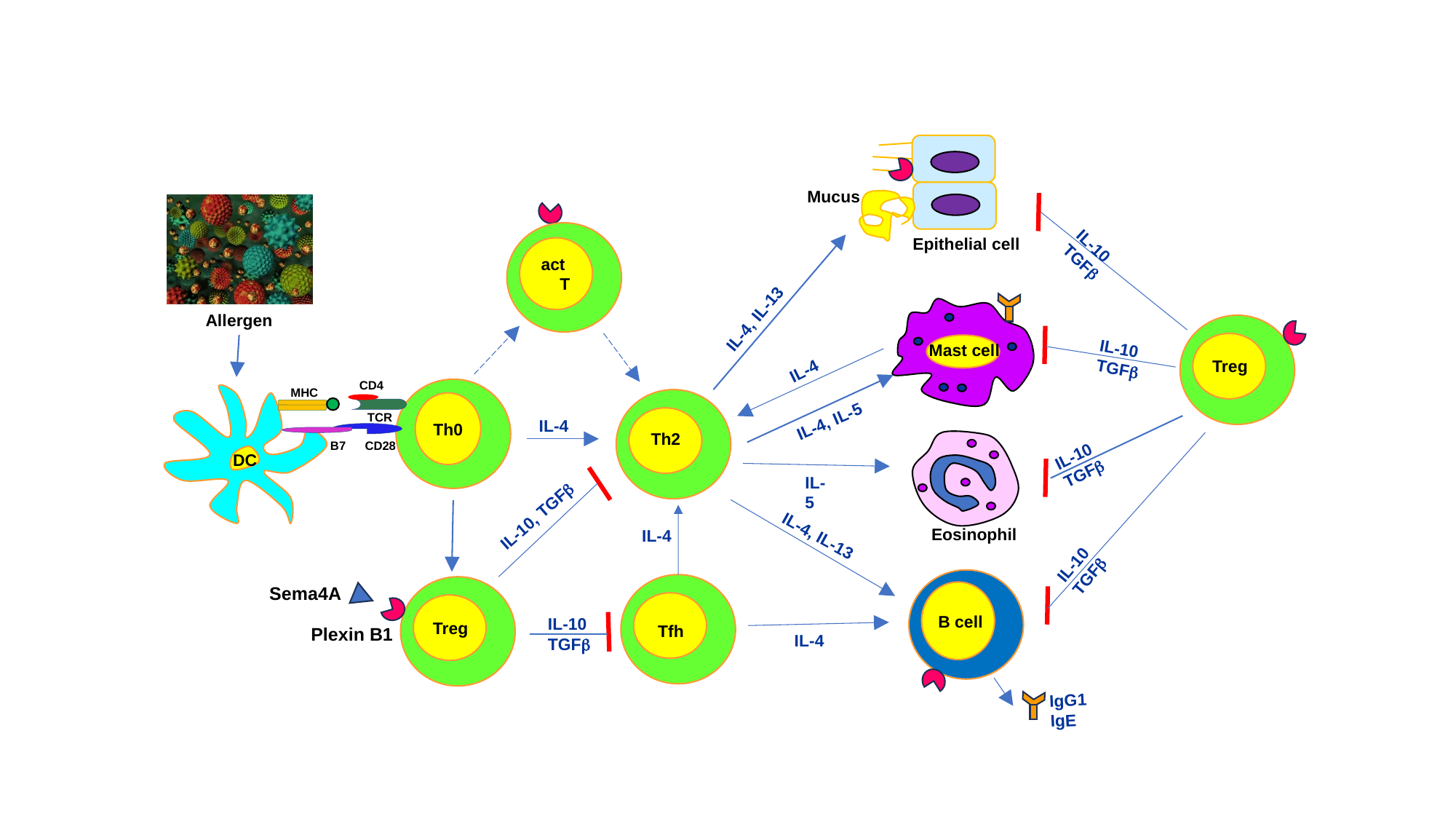

Mucus
Epithelial cell
IL-10
TGFb
act T
IL-4, IL-13
Allergen
IL-10
TGFb
Mast cell
Treg
IL-4
CD4
MHC
Th0
TCR
IL-4, IL-5
IL-4
Th0
Th2
B7
CD28
IL-10
TGFb
DC
IL-5
IL-10, TGFb
Eosinophil
IL-4
IL-4, IL-13
IL-10
TGFb
Sema4A
B cell
IL-10
TGFb
Treg
Tfh
Plexin B1
IL-4
IgG1
IgE

## Slide 2
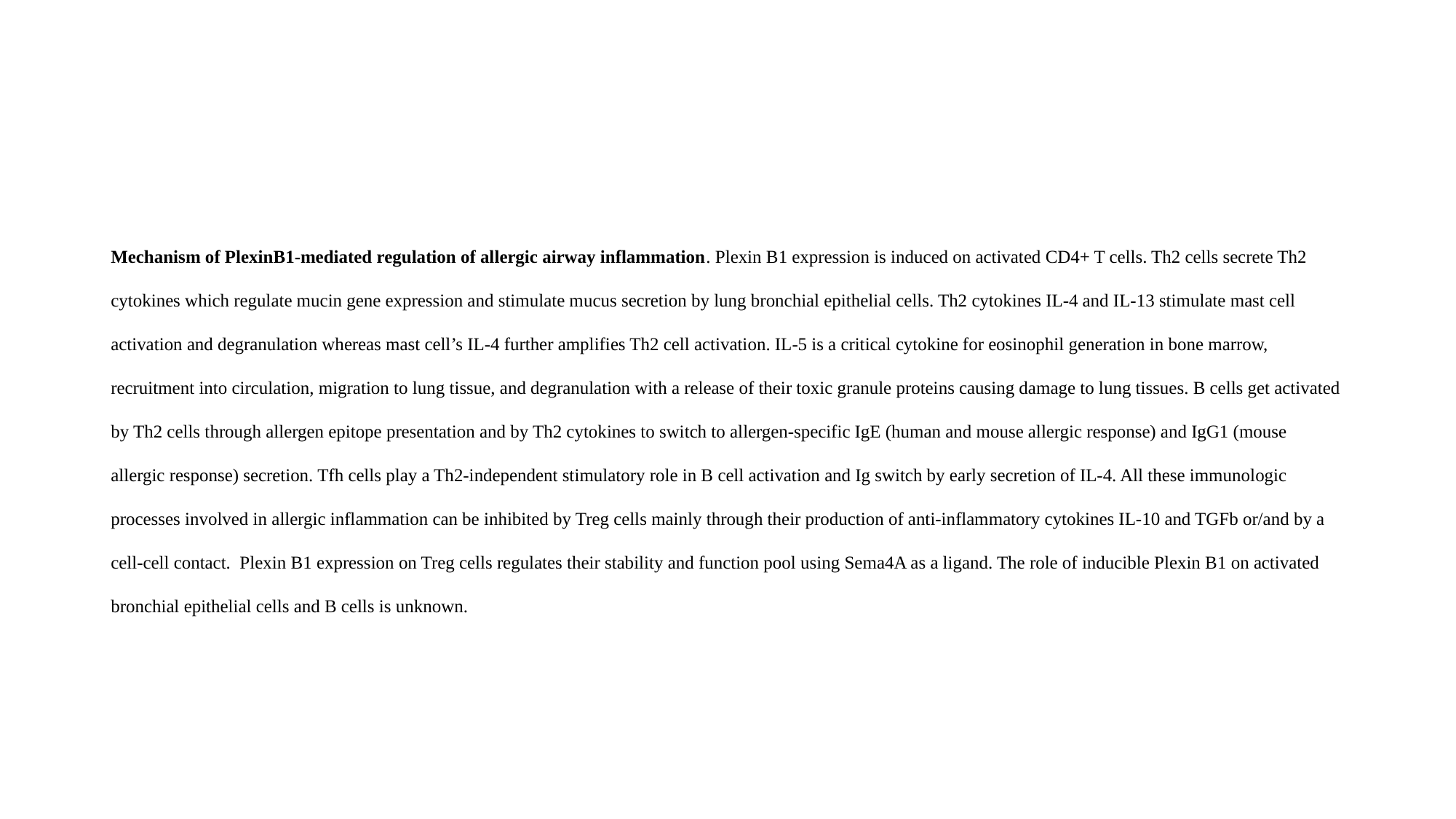

Mechanism of PlexinB1-mediated regulation of allergic airway inflammation. Plexin B1 expression is induced on activated CD4+ T cells. Th2 cells secrete Th2 cytokines which regulate mucin gene expression and stimulate mucus secretion by lung bronchial epithelial cells. Th2 cytokines IL-4 and IL-13 stimulate mast cell activation and degranulation whereas mast cell’s IL-4 further amplifies Th2 cell activation. IL-5 is a critical cytokine for eosinophil generation in bone marrow, recruitment into circulation, migration to lung tissue, and degranulation with a release of their toxic granule proteins causing damage to lung tissues. B cells get activated by Th2 cells through allergen epitope presentation and by Th2 cytokines to switch to allergen-specific IgE (human and mouse allergic response) and IgG1 (mouse allergic response) secretion. Tfh cells play a Th2-independent stimulatory role in B cell activation and Ig switch by early secretion of IL-4. All these immunologic processes involved in allergic inflammation can be inhibited by Treg cells mainly through their production of anti-inflammatory cytokines IL-10 and TGFb or/and by a cell-cell contact. Plexin B1 expression on Treg cells regulates their stability and function pool using Sema4A as a ligand. The role of inducible Plexin B1 on activated bronchial epithelial cells and B cells is unknown.
